# *Ophiocordyceps sinensis* preparations combined with the renin–angiotensin system inhibitor for diabetic kidney disease treatment: an umbrella review of systematic reviews and network meta-analysis

**DOI:** 10.3389/fphar.2024.1360633

**Published:** 2024-04-22

**Authors:** Xue Xue, Xin-Yan Jin, Xing-Lan Ye, Ke-Ying Li, Jia-Xuan Li, Xue-Han Liu, Juan Bai, Qiang Liu, Bing-Rui Zhang, Xin-Rong Zou, Jun Yuan, Chun-Li Lu, Fang-Fang Zhao, Jian-Ping Liu, Xiao-Qin Wang

**Affiliations:** ^1^ Affiliated Hospital of Hubei University of Chinese Medicine, Wuhan, China; ^2^ Centre for Evidence-Based Chinese Medicine, Beijing University of Chinese Medicine, Beijing, China; ^3^ School of Clinical Traditional Chinese Medicine, Hubei University of Chinese Medicine, Wuhan, China; ^4^ Hubei Key Laboratory of Theory and Application Research of Liver and Kidney in Traditional Chinese Medicine, Affiliated Hospital of Hubei University of Chinese Medicine, Hubei Provincial Hospital of Traditional Chinese Medicine, Wuhan, China; ^5^ Dongzhimen Hospital, Beijing University of Chinese Medicine, Beijing, China; ^6^ Department of Nephrology, Renmin Hospital of Wuhan University, Wuhan University, Wuhan, China; ^7^ Traditional Chinese Medicine Research Institute, Guangdong Pharmaceutical University, Guangzhou, China; ^8^ Xiyuan Hospital, China Academy of Chinese Medical Sciences, Beijing, China

**Keywords:** diabetic kidney disease, *Ophiocordyceps sinensis* preparations, renin–angiotensin system inhibitor, umbrella review, network meta-analysis

## Abstract

**Aims:**

This study aimed to synthesize the evidence of the comparative effectiveness and safety of *Ophiocordyceps sinensis* (*OS*) preparations combined with renin–angiotensin system inhibitors (RASi) for diabetic kidney disease (DKD).

**Methods:**

Eight databases were searched from their inception to May 2023. Systematic reviews (SRs) of *OS* preparations combined with RASi for DKD were identified. Randomized controlled trials (RCTs) from the included SRs and additional searching were performed for data pooling. Cochrane risk-of-bias 2 (RoB 2) tool and AMSTAR 2 were used to evaluate the methodological quality of RCTs and SRs, respectively. A Bayesian network meta-analysis was performed to compare the add-on effect and safety of *OS* preparations for DKD. The certainty of evidence was graded using the Grading of Recommendations, Assessment, Development, and Evaluation (GRADE) approach.

**Results:**

Fourteen SRs were included, whose methodological quality was assessed as high (1/14) or critically low (13/14). After combining additional searching, 157 RCTs were included, involving 13,143 participants. The quality of the RCTs showed some concerns (155/157) or high risk (2/157). Jinshuibao capsules and tablets, Bailing capsules and tablets, and Zhiling capsules were evaluated. Compared to RASi, adding either of the *OS* capsular preparations resulted in a decreased 24-h urinary total protein levels. *OS* preparations ranked differently in each outcome. Jinshuibao capsules plus RASi were beneficial in reducing urinary protein, serum creatinine, serum urea nitrogen, and blood glucose levels, with moderate-certainty evidence. No serious adverse events were observed after adding *OS* to RASi.

**Conclusion:**

Combining *OS* capsular preparations with RASi appeared to be associated with decreased urinary total protein levels in DKD patients. Further high-quality studies are needed to confirm.

**Systematic Review Registration:**

INPASY202350066.

## 1 Introduction

The prevalence of diabetes mellitus (DM) has increased rapidly over the past decades worldwide (Lovic et al., 2020). As one of the common and serious microvascular complications of diabetes, the prevalence of diabetic kidney disease (DKD) has also increased significantly (Tuttle et al., 2022). Although the spectrum for the etiology of chronic kidney disease (CKD) in China differs from that in Western countries (Wang and He, 2021), such a trend has also been found in recent studies. The results of the Sixth China Chronic Disease and Risk Factor Surveillance showed that the prevalence of CKD associated with DM increased proportionally, despite the observed decreasing trend in the overall prevalence of CKD in China (Zhang et al., 2016; Wang et al., 2023). DKD patients have a poor prognosis compared to non-DKD patients. DKD substantially increases the risk of kidney failure and cardiovascular events (Tuttle et al., 2022). Therefore, DKD has become a global public health problem with a significant disease burden.

At present, the basic prevention and treatment therapies for DKD are lifestyle change and risk factor control, including exercise, nutrition, smoking cessation, glycemic control, blood pressure control, and lipid management (KDIGO, 2022). In addition, renin–angiotensin system inhibitors (RASi) have a renal protective effect independent of decreasing blood pressure (Leoncini et al., 2020). Previously, only RASi with multidisciplinary therapy were effective for DKD. Recent studies on some newly developed hypoglycemic drugs suggested that they may have a potential renal protective effect, such as sodium–glucose cotransporter 2 (SGLT-2) inhibitors and glucagon-like peptide-1 (GLP−1) receptor agonists (Kristensen et al., 2019; Zelniker et al., 2019). Despite the remarkable progress, there is still a substantial residual risk of disease progression with existing therapies (Tuttle et al., 2022). In addition, side effects and the requirement for the estimated glomerular filtration rate (eGFR) in the indications of drug use still limit the clinical applications of these drugs. Overall, DKD is undoubtedly a medical challenge all over the world.

*Ophiocordyceps sinensis* (*OS*), popularly known as caterpillar mushroom, is a non-toxic, medicinal fungus growing in the Himalayan hills in Nepal, India, and Tibet, China (Singh et al., 2018). *OS* contains cordycepin, carbohydrate d-mannitol, vitamin B_12_, six essential amino acids, and unsaturated fatty acids (Singh et al., 2018). A number of scientific research studies have indicated that *OS* has anti-inflammatory, anticancer, antidiabetic, analgesic, antioxidant, anti-allergic, and anti-obesity effects (Olatunji et al., 2018). According to the traditional Chinese medicine (TCM) theory, *OS* has the function of securing the essence and strengthening Qi, reinforcing the lung and kidney. In particular, *OS* has a good therapeutic effect on lung and renal diseases (Zhang et al., 2015; Wang et al., 2021). Because wild *OS* is rare and expensive, it can no longer meet the market demand. As the fermentation technology of medicinal strains isolated from *OS* is becoming increasingly mature, preparations composed of artificially fermented *OS* powder have become more and more common in clinical applications. At present, the main *OS* preparations of Chinese patent medicine products include Jinshuibao capsule/tablet (species name: *Paecilomyces hepiali chen*), Bailing capsule/tablet/granule (*Hirsutella sinensis Liu, Guo, Yu-et Zeng*), Zhiling capsule (*Mortierella sp.*), Xinganbao capsule (*Gliocladium roseum (link) Thom*), Yong Chong Cao capsule (*Cordyceps militaris L. Link*), and Ningxinbao capsule (*Cephalosporium sinensis Chen. sp. nov*) (Zheng et al., 2011; Wang et al., 2016). Even if derived from the same species name, the preparations of different dosage forms are not exactly the same. A study comparing the amino acid fingerprint and common peak map of Jinshuibao capsules and tablets clarified the differences between the two, which may be used as the basis for distinguishing different preparations (Zhao, 2019).

Accumulating research on *OS* preparations combined with RASi for DKD treatment exists, but there is a lack of summary of the overall evidence and comparison between different *OS* preparations. The objective of this study is to evaluate the methodological quality of existing systematic reviews (SRs) and compare the effectiveness and safety of different *OS* preparations (including different dosage forms) when used in combination with RASi in DKD patients. The umbrella review and network meta-analysis (NMA) approaches were used to provide comprehensive evidence and reference for clinical rational drug use.

## 2 Methods

### 2.1 Protocol and registration

This study followed the methodological process of the Joanna Briggs Institute for an “umbrella review” (Aromataris et al., 2015; Aromataris et al., 2020). The protocol of this review was specified in advance and registered on the International Platform of Registered Systematic Review and Meta-analysis Protocols (INPLASY.COM) (Registration number: 202350066). The study was reported according to the Preferred Reporting Items for Overviews of Reviews (PRIOR) statement (Gates et al., 2022).

### 2.2 Eligibility criteria

#### 2.2.1 Types of participants

All participants met the diagnostic criteria for DKD (Kopel et al., 2019; Expert Group of Chinese Society of Nephrology, 2021). None of the participants entered renal replacement therapies, including peritoneal dialysis, hemodialysis, and kidney transplant. There were no restrictions on age, sex, race, stage of disease, or source of cases.

#### 2.2.2 Types of interventions

In addition to basic treatment (BT), any *OS* preparation added was included in the experimental group. BT referred to exercise, nutrition, smoking cessation, glycemic control, blood pressure control, lipid management, and RASi use.

#### 2.2.3 Types of comparisons

The control group involved “BT alone” or “BT plus placebo” or “BT plus different *OS* preparation.” There is no restriction on the form or dosage of *OS* preparation.

#### 2.2.4 Types of outcomes

We specified the relative importance of the outcomes according to the Grades of Recommendation, Assessment, Development, and Evaluation (GRADE) approach (Guyatt et al., 2011) by referring to the clinical practice guideline of KDIGO (2022) and consulting clinical doctors. SRs that reported at least one of the following outcomes were included in this study. Critical outcomes included (Lovic et al., 2020) end-point events including all-cause mortality, doubling of the serum creatinine (Scr) level, decrease in the eGFR by more than 50% from the baseline level, or entry into renal replacement therapies (Tuttle et al., 2022); albuminuria progression including the onset of albuminuria, moderately increased (formerly known as microalbuminuria) to severely increased albuminuria (formerly known as macroalbuminuria) (Wang and He, 2021); and major adverse cardiovascular and cerebrovascular events (MACCEs) including cardiovascular mortality, acute myocardial infarction, hospitalization for congestive heart failure, intraparenchymal hemorrhage, subarachnoid hemorrhage, or cerebral infarction. Important but not critical outcomes involved urine protein testing, renal function testing, and adverse events (AEs). The specific outcomes on urine protein testing included 24-h urinary total protein (24-h UTP), urinary albumin excretion rate (UAER), and urinary albumin/creatinine ratio (UACR). The specific outcomes on renal function included Scr, serum urea nitrogen (SUN), and eGFR. Other outcomes were fasting plasma glucose (FPG), glycated hemoglobin A1c (HbA1c), and patient-reported symptoms.

#### 2.2.5 Types of studies

All the SRs and meta-analysis of randomized controlled trials (RCTs) were included in this umbrella review. All RCTs included in SRs and RCTs obtained after additional retrieval were used for network meta-analysis after overlapping was excluded.

### 2.3 Search strategy

We searched the following Chinese and English databases from their inception to May 2023. Chinese databases included the China National Knowledge Infrastructure (CNKI), Wanfang, Chinese Science and Technology Journal Database (VIP), and SINOMED database. English databases included PubMed, Embase, the Cochrane Library, and the Web of Science. Two international platforms of registered SR and meta-analysis protocols including INPLASY and PROSPERO were also searched. No language or publication type is imposed. Additional retrieval for RCTs was conducted from the date of the most recently searched data on published SRs to 15 May 2023. The retrieval strategies for SRs and RCTs are specified in Supplementary Table S1.

### 2.4 Study selection and data extraction

Two authors (X Xue and KY Li) independently screened the eligible SRs and then screened the RCTs from the SRs for inclusion in the network meta-analysis. Disagreements of selection and data extraction were resolved through discussions with the corresponding author.

Two authors (KY Li and XL Ye) extracted the data, and two authors (X Xue and JX Li) checked those. The following information was extracted: first author, publication year, design and the number of included studies, participants, intervention/comparison measures, outcomes, and methodological quality evaluation information.

For the network meta-analysis, the following items were collected from the RCTs: study identification, characteristics of participants, trade names of *OS* preparations, daily dosage, treatment duration, outcomes, and adverse events.

### 2.5 Methodological quality assessment

The AMSTAR 2 tool (Shea et al., 2017) was applied to appraise the methodological quality of the included SRs by two authors (X Xue and XY Jin) independently. Two authors (X Xue and XY Jin) assessed the quality of RCTs using the risk-of-bias 2 (RoB 2) tool from the Cochrane Library (Sterne et al., 2019). Any disagreements were resolved by discussion with the corresponding author.

### 2.6 Data synthesis and statistical methods

R software version 4.2.1 was used for analysis. The pooled results were presented as the mean difference (MD) or risk ratio (RR) with 95% confidence intervals (CIs). A *p*-value of <0.05 was considered statistically significant. The Bayesian network meta-analysis was performed using Markov chain Monte Carlo (MCMC) simulation (Phillippo et al., 2020). A potential scale reduction factor (PSRF) value, the median, and 97.5% of the PSRF value close to or equal to 1 indicate good convergence of the MCMC algorithm (1 < PSRF ≤ 1.05) (Tunaru, 2002; Valkenhoef et al., 2012). If the PSRF value is not in this range, the iteration continues manually until the PSRF value reaches the range standard. An evidence network is performed according to each outcome of our interest. The data are analyzed by adjusting the indirect comparison approach if closed loops are unavailable, while the mixed treatment comparison approach is performed when one or more closed loops are available. For the local inconsistency estimate based on the existence of one or more closed loops, the consistency of direct and indirect comparisons is assessed by the node-split model. The consistency model is adopted if the *p*-value is > 0.05, whereas the inconsistent model is used for analysis (Dias et al., 2010; Van Valkenhoef et al., 2016). The results of the NMA showed that the ranking probability plot and the surface under the cumulative ranking (SUCRA) plot are generated and sorted by dominance. The value of SUCRA is between 0 and 1. When the SUCRA value is 1, it indicates that the intervention is absolutely effective. The effect of interventions can be ranked based on the value of SUCRA.

If closed loops are available, both the consistency model and unrelated mean effects model are adopted as sensitivity analysis to explore the robustness of the results. Regarding the choice of random-effects model or fixed-effects model, we refer to values of DIC and Dbar. If the difference in the DIC value between models is greater than 5, the model with the smaller DIC is selected. When the difference is between 3 and 5, the model with the lower Dbar value is adopted; if the difference is less than 3, the fixed-effects model is used. If more than 10 trials are included, counter-enhanced funnel plots combined with the trim and fill method are applied to evaluate publication bias.

### 2.7 Assessment of the certainty of evidence

The certainty of the evidence was assessed by RCTs after excluding overlapping. Two authors (X Xue and XY Jin) independently assessed direct evidence, indirect evidence, and combined evidence for outcomes using the GRADE approach (Atkins et al., 2004) if closed loops were available in the network. When closed loops were unavailable, the direct and indirect evidence for significant outcomes was assessed.

## 3 Results

### 3.1 Characteristics of included reviews

**FIGURE 1:**
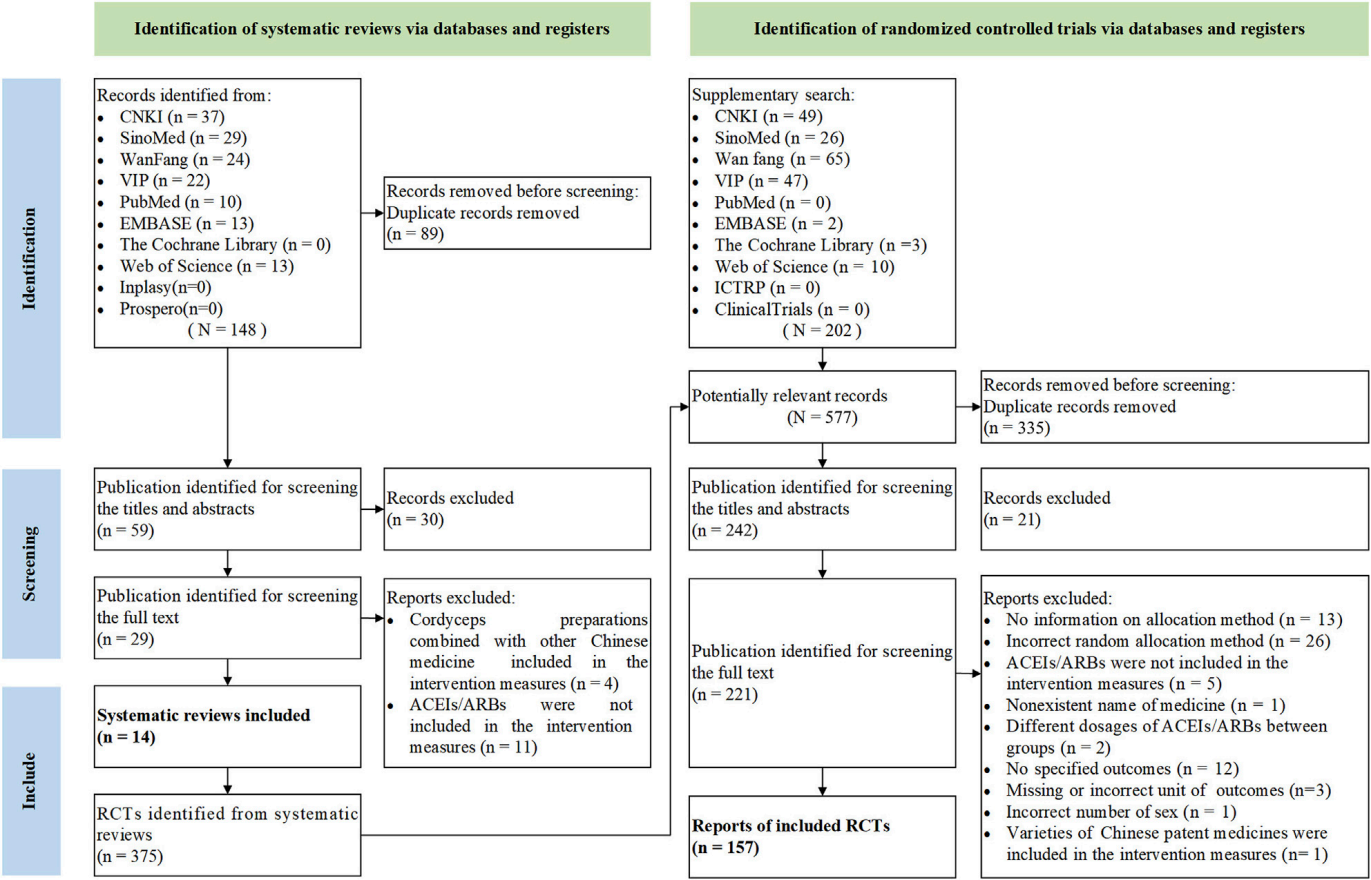
Flow diagram of search and selection.

A total of 148 citations were retrieved from the initial searches, and 30 citations were screened out by reading the titles and abstracts. After scanning the full texts, 15 SRs were excluded (Zhang et al., 2012; He and Ge, 2012; Huang et al., 2012; Mao et al., 2012; Tang et al., 2013; Wang and Duan, 2013; Ji et al., 2014; Chen et al., 2017; Jing et al., 2017; Wen et al., 2018; Liu et al., 2020; Sheng et al., 2020; Gao et al., 2021; Su et al., 2021; Yu et al., 2022), and 14 SRs were included finally (Tang et al., 2013; Duan and Li, 2015; Luo et al., 2015; Cai, 2016; Li, 2016; Lu et al., 2018; Zhang, 2018; Huang et al., 2019; Li and Xu, 2019; Lian et al., 2020; Gong et al., 2021; Zhou et al., 2021; Li et al., 2022; Yan et al., 2023). The screening and selection process of the literature is shown in Figure 1. The list of exclusions during the full-text screening process is presented in Supplementary Table S2. A total of five varieties of *OS* preparations were involved in the 14 SRs, namely, Bailing capsule (BLC), Bailing tablet (BLT), Jinshuibao capsule (JSBC), Jinshuibao tablet (JSBT), and Zhiling capsule (ZLC).

Fourteen SRs were published between 2013 and 2023, and all have been published in full text. Ten SRs were published in Chinese and four in English. Fourteen SRs were performed in China. The number of RCTs in the included SRs ranged from 4 to 60, and the total sample size ranged from 241 to 4,562 participants. Types of intervention measures were BT combined with *OS* preparations. Types of control measures included BT alone. The total course of treatment in 14 SRs ranged from 2 to 48 weeks. Furthermore, we did not find the critical outcomes in all the included SRs. Among the important outcomes, we found 6 relevant measures, namely, UAER (13/14, 92.86%), 24-h UTP (12/14, 85.71%), UACR (3/14, 21.43%), Scr (13/14, 92.86%), SUN (11/14, 78.57%), and adverse events (8/14, 57.14%). Regarding other outcomes, FPG was reported in 10 SRs (10/14, 71.43%), and HbA1c was reported in 7 SRs (7/14, 50.00%). No review mentioned patient-reported symptoms. The general characteristics of each review are presented in Table 1.

**TABLE 1:** Characteristics of each included systematic review.

Study ID	Included RCTs (n)	Sample size (total, E/C)	Stage of DKD patients	Intervention		Outcomes	Risk assessment tool
				Experimental group	Control group		
Tang L 2013	24	2,653 (1,340/1,313)	Unclear	BLC/ZLC/JSBC + BT	BT (irbesartan/valsartan/losartan/telmisartan)	(1)(2)(3)	Cochrane RoB
Duan R 2015	15	1,095 (546/549)	Unclear	JSBC + BT	BT (candesartan cilexetil/olmesartan medoxomil/valsartan/losartan/losartan potassium/telmisartan/irbesartan)	(1)(2)(3)(4)(5)	Cochrane RoB
Luo Y 2015	60	4,288 (2,163/2,165)	Ⅳ	JSBC + BT	BT (benazepril/valsartan/perindopril/irbesartan/enalapril/candesartan/olmesartan/telmisartan/losartan/fosinopril)	(1)(2)(3)(4)(6)(7)(8)	Jadad
Cai T 2016	15	1,085 (542/543)	Ⅲ	BLC + BT	BT (benazepril/enalapril/fosinopril sodium/captopril) + placebo	(1)(3)(4)(7)(8)	Cochrane RoB
Li GD 2016	4	241 (121/120)	Unclear	ZLC + BT	BT (irbesartan/losartan potassium/valsartan)	(1)(2)(7)	Cochrane RoB
Zhang Y 2018	12	1,124 (561/563)	Ⅰ–Ⅲ	BLC + BT	BT (irbesartan/losartan/valsartan/telmisartan)	(1)(2)(3)(4)(6)(7)(8)	Modified Jadad
Lu Q 2018	26	2,198 (1,067/1,131)	Ⅲ	JSBC + BT	BT (valsartan/irbesartan/candesartan cilexetil/telmisartan/losartan/olmesartan medoxomil/losartan potassium/candesartan)	(1)(2)(3)(4)(5)(6)(7)(8)	Cochrane-RoB
Huang YL 2019	31	2,434(1,229/1,205)	Unclear	BLC + BT	BT (benazepril/valsartan/irbesartan/losartan/telmisartan/enalapril/candesartan cilexetil)	(1)(2)(3)(6)(7)(8)	Cochrane RoB and Jadad
Lian XY 2020	14	1,067 (538/529)	Unclear	JSBC + BT	BT (perindopril tert-butylamine/benazepril/enalapril/benazepril hydrochloride)	(1)(2)(3)(4)(6)	Cochrane RoB
Li YB 2019	51	3,955 (1992/1963)	Unclear	JSBC + BT	BT (benazepril hydrochloride/benazepril/fosinopril/perindopril/enalapril/olmesartan medoxomil/irbesartan/candesartan cilexetil/candesartan/losartan/losartan potassium/telmisartan)	(1)(2)(3)(4)(6)(7)(8)	Cochrane RoB
Gong L 2021	10	782 (385/397)	Ⅲ–Ⅳ	BLC + BT	BT (valsartan)	(2)(3)(4)(6)	Cochrane RoB
Zhou XM 2021	48	4,562 (2,281/2,281)	Unclear	BLC/BLT/JSBC/JSBT/ZLC + BT	BT (benazepril/valsartan/irbesartan)	(1)(2)(3)(4)(6)(7)(8)	Cochrane RoB and modified Jadad
Li XJ 2022	12	930 (465/465)	Unclear	JSBC + BT	BT (irbesartan)	(1)(2)(4)(7)	Cochrane RoB
Yan GC 2023	38	3,167 (1,601/1,566)	Unclear	BLC/BLT/JSBC/JSBT/ZLC/NXBC/YCCC + BT	BT (benazepril/valsartan/irbesartan1/telmisartan/enalapril/losartan/candesartan)	(1)(2)(3)(4)(5)(6)(7)	Cochrane RoB

Note: (1) Urinary albumin excretion rate; (2) serum creatinine; (3) 24-h urinary total protein; (4) serum urea nitrogen; (5) urinary albumin/creatinine ratio; (6) adverse events; (7) fasting plasma glucose; and (8) glycated hemoglobin A1c. Abbreviations: RCTs, randomized controlled trials; E/C, experimental group/control group; DKD, diabetic kidney disease; BT, basic treatment; RoB, risk of bias; NR, not reported; BLC, Bailing capsule; BLT, Bailing tablet; JSBC, Jinshuibao capsule; JSBT, Jinshuibao tablet; ZLC, Zhiling capsule; NXBC, Ningxinbao capsule; YCCC, Yongchongcao capsule.

### 3.2 Methodological quality evaluation of included reviews

We appraised the methodological quality of the included SRs using AMSTAR 2. The results indicated that 1 SR was “high,” and 13 SRs were “critically low” in methodological quality, as shown in Supplementary Table S3.

### 3.3 Characteristics of included RCTs

A total of 375 primary RCTs were included in the SRs. We conducted an additional search of 264 RCTs. After 397 overlapping RCTs were excluded and further screened according to the eligibility criteria, a total of 157 eligible RCTs were included in the final analysis (Jia, 1999; Cao et al., 2007; Chen, 2007; Chen, 2009; Cui et al., 2009; Lei et al., 2009; Li and Liu, 2009; Chen et al., 2010; Guan et al., 2010; Hong, 2010; Huang et al., 2010; Chen, 2011; Cao, 2012; He and Lu, 2012; Hu and Wei, 2012; Gao and Wei, 2013; Ding, 2014; Cao, 2015; Diao, 2015; Chen et al., 2016; Dai, 2016; Hu, 2016; Huang and Zhou, 2016; Jin, 2016; Chen, 2017; Feng, 2017; He, 2017; Hou et al., 2017; Lei et al., 2017; Gao, 2018; Hu et al., 2018; Dai, 2019; Hu, 2019; Cheng, 2021; Du, 2021; Guan et al., 2021; Han, 2021; Jiang, 2021; Ding et al., 2022; Dong, 2022; Huang et al., 2022; Li and Lv, 2011; Li et al., 2019; Li, 2012; Li, 2010; Li, 2012; Li, 2017; Li, 2019; Li, 2023; Lin, 2009; Lin, 2016; Liu and Jian, 2020; Liu and Li, 2011a; Liu and Li, 2011b; Liu and Zhang, 2017; Liu et al., 2016a; Liu et al., 2016b; Liu, 2010; Liu, 2011; Liu, 2011; Liu, 2017; Liu, 2019; Liu, 2019; Long, 2014; Lou et al., 2010; Lu and Shi, 2020; Lu, 2010; Luo et al., 2011; Luo et al., 2018; Lv et al., 2006; Lv, 2012; Lv, 2012; Lv, 2014; Lv, 2022; Ma et al., 2011; Ma, 2017; Ma, 2018; Pan and Shang, 2016; Qiu et al., 2019; Ren and Hu, 2020; Ren et al., 2020; Shan, 2012; Shen and Chen, 2011; Shen et al., 2018; Shen, 2012; Shen et al., 2021; Shi, 2010; Song and Ning, 2017; Song et al., 2009; Sun et al., 2012; Tang, 2011; Tang, 2016; Tao and Zhou, 2009; Tian, 2019; Wang and He, 2015; Wang and Qi, 2018; Wang and Yuan, 2008; Wang and Zheng, 2010; Wang et al., 2009; Wang et al., 2020; Wang et al., 2020; Wang et al., 2020; Wang, 2007; Wang, 2011; Wang, 2011; Wang, 2012; Wei, 2010; Wu and Pan, 2016; Wu et al., 2014; Wu et al., 2019; Wu, 2008; Wu, 2012; Xiao, 2021; Xie and Mo, 2021; Xie, 2013; Yan and Wang, 2005; Xue, 2009; Yi et al., 2009; Zhang and Lu, 2009; Zeng and Zhang, 2010; Yang and Yang, 2011; Zhang, 2011; Zheng, 2011; Zhang et al., 2012; Ye et al., 2012; Yu, 2012; Zhou, 2012; Yang, 2013; Yu et al., 2013; Zhou et al., 2013; Zeng, 2014; Zhang et al., 2014; Zhang and Zuo, 2014; Zong and Ding, 2014; Xu, 2015; Zeng, 2015; Zhou et al., 2015; Zhu and Qiu, 2015; Yang et al., 2016; Zhan et al., 2016; Zhang, 2016; Yang et al., 2017; Yuan, 2017; Zhang et al., 2017; Xu, 2018; Yang, 2019; Zhang and Zhang, 2019; Xu, 2021; Yu et al., 2021; Zhang, 2021; Yu, 2022), involving 13,143 participants. Although we tried our best to contact the corresponding authors by email or phone in order to acquire the questionable information about the characteristics of RCTs, only three authors responded. Details are given in Supplementary Table S4. The recruitment timeline ranged from 1999 to 2023. All 157 trials were designed as two-armed RCTs (157/157, 100%). In addition, 151 trials were performed at a single center (151/157, 96.18%), while 1 trial was performed at a multi-center (1/157, 0.64%). The sample size of the included trials ranged from 36 to 252 participants, with an average of 84. Sex information was reported in 152 trials (152/157, 96.82%), in which 6,971 people were men and 5,682 were women. The participants’ age ranged from 32 to 85 years, while 9 trials did not report any age information (9/157, 5.73%). The comparisons of included trials were all BT plus *OS* preparations *versus OS* preparations, involving BLC, BLT, JSBC, JSBT, and ZLC. The intervention duration was arranged from 2 weeks to 12 months, with 12 weeks as common. None of the RCTs reported the critical outcomes. All 157 trials reported important but not critical outcomes (157/157, 100%), among which 127 trials involved urine protein testing (127/157, 80.89%), 118 trials involved renal function testing (118/157, 75.16%), and only 48 trials involved adverse events (48/157, 30.57%). Only one trial reported a follow-up (1/157, 0.64%). Funding sources were reported in 15 trials (15/157, 9.55%), with no funding from pharmaceutical companies. The main characteristics of the included primary trials are given in Supplementary Table S5.

### 3.4 Methodological quality evaluation of included randomized controlled trials

The risk of bias in the RCTs was assessed by the randomization of processes, deviations from intended interventions, missing outcome data, measurement of the outcome, and selection of the reported result using the RoB 2 tool. The results showed that all trials were assessed as having “some concerns” in the randomization process domain, which resulted in the overall methodological quality of the RCTs being “some concern” (155/157, 98.73%). As for missing outcome data and measurement of the outcome domains, all trials were assessed as “low risk.” However, two trials showed “high risk” in deviation from the intended intervention domain, and the overall methodological quality was assessed as “high risk” (2/157, 1.27%). The risk of bias in each included trial is given in Supplementary Figure S1.

### 3.5 Effects of interventions

The Bayesian network model was constructed using gemtc, BUGSnet, and multinma packages in R software. The trace and density plots and Brooks–Gelman–Rubin diagnostic plots show that the model convergence degree of each outcome was satisfactory (Supplementary Figure S2). Therefore, the Bayesian model established in this study can effectively predict the results.

#### 3.5.1 Important but not critical outcome: 24-h urinary total protein

**FIGURE 2:**
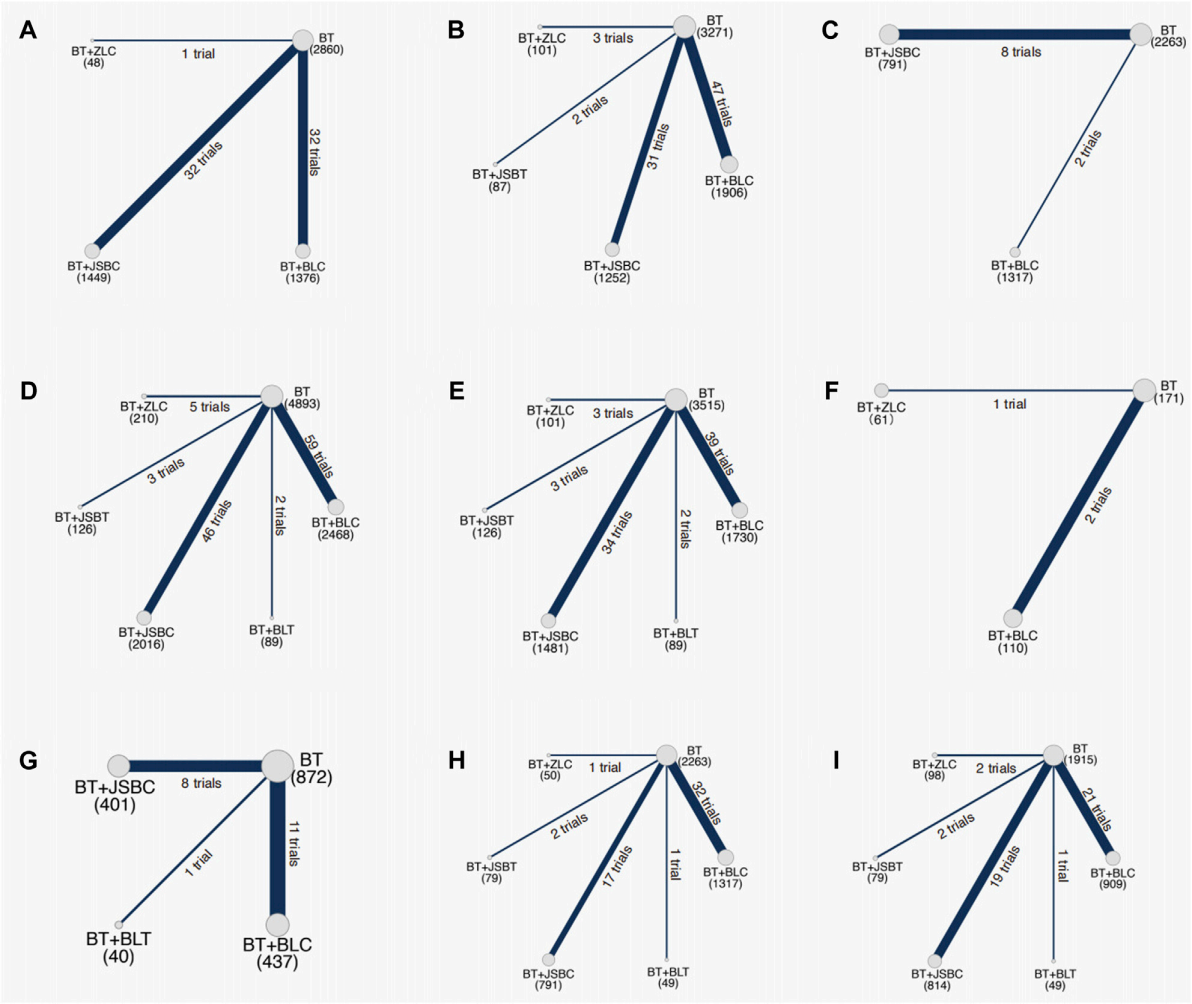
Network evidence on outcomes. Note: **(A)** 24-h urinary total protein; **(B)** urinary albumin excretion rate; **(C)** urinary albumin/creatinine ratio; **(D)** serum creatinine; **(E)** serum urea nitrogen; **(F)** estimated glomerular filtration rate; **(G)** adverse events; **(H)** fasting plasma glucose; and **(I)** glycated hemoglobin A1c. BT, basic treatment; BLC, Bailing capsule; BLT, Bailing tablet; JSBC, Jinshuibao capsule; JSBT, Jinshuibao tablet; and ZLC, Zhiling capsule.

**FIGURE 3:**
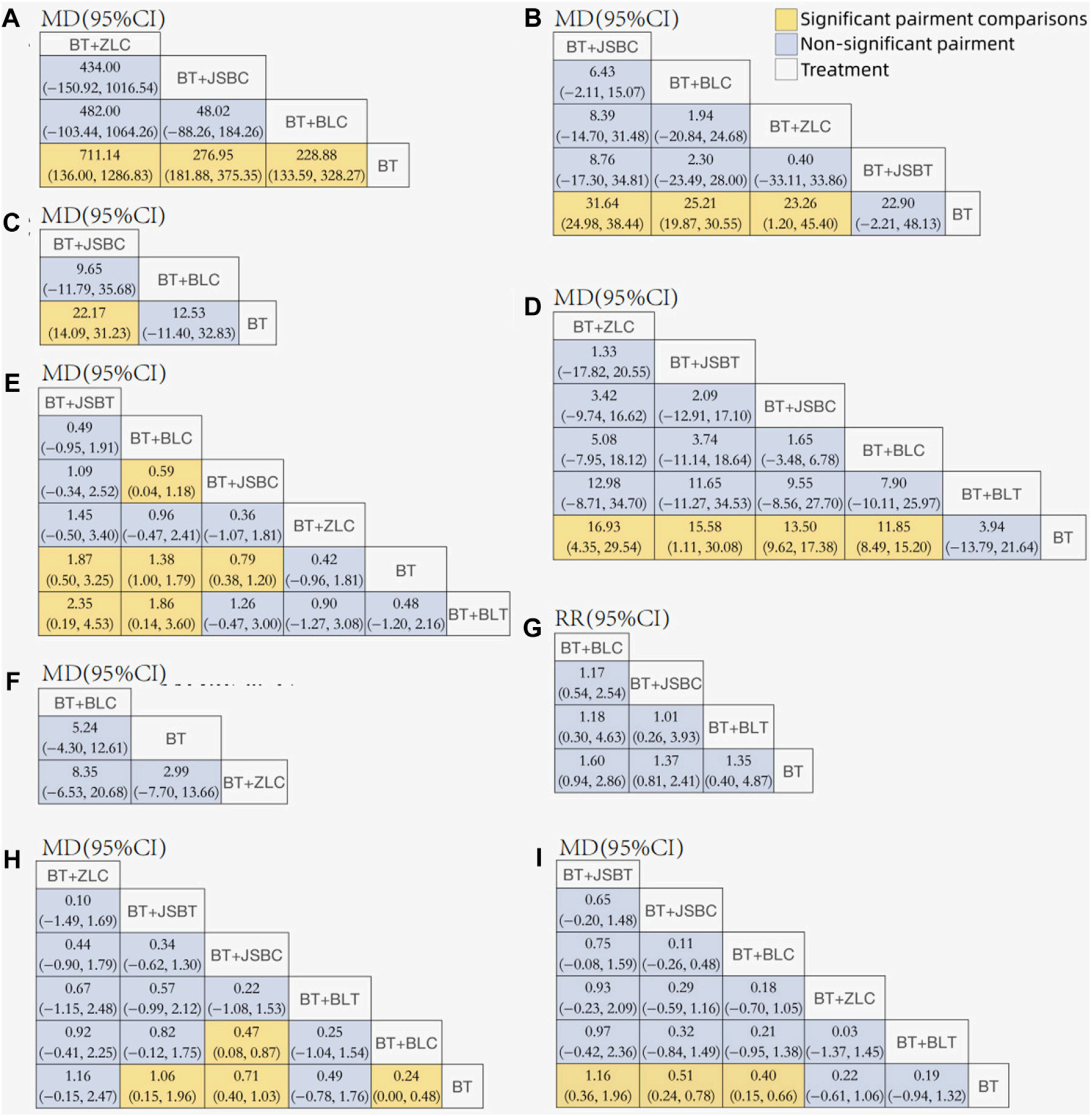
League tables for comparison between each intervention. Note: **(A)** 24-h urinary total protein; **(B)** urinary albumin excretion rate; **(C)** urinary albumin/creatinine ratio; **(D)** serum creatinine; **(E)** serum urea nitrogen; **(F)** estimated glomerular filtration rate; **(G)** adverse events; **(H)** fasting plasma glucose; and **(I)** glycated hemoglobin A1c. BT, basic treatment; BLC, Bailing capsule; BLT, Bailing tablet; JSBC, Jinshuibao capsule; JSBT, Jinshuibao tablet; and ZLC, Zhiling capsule.

**FIGURE 4:**
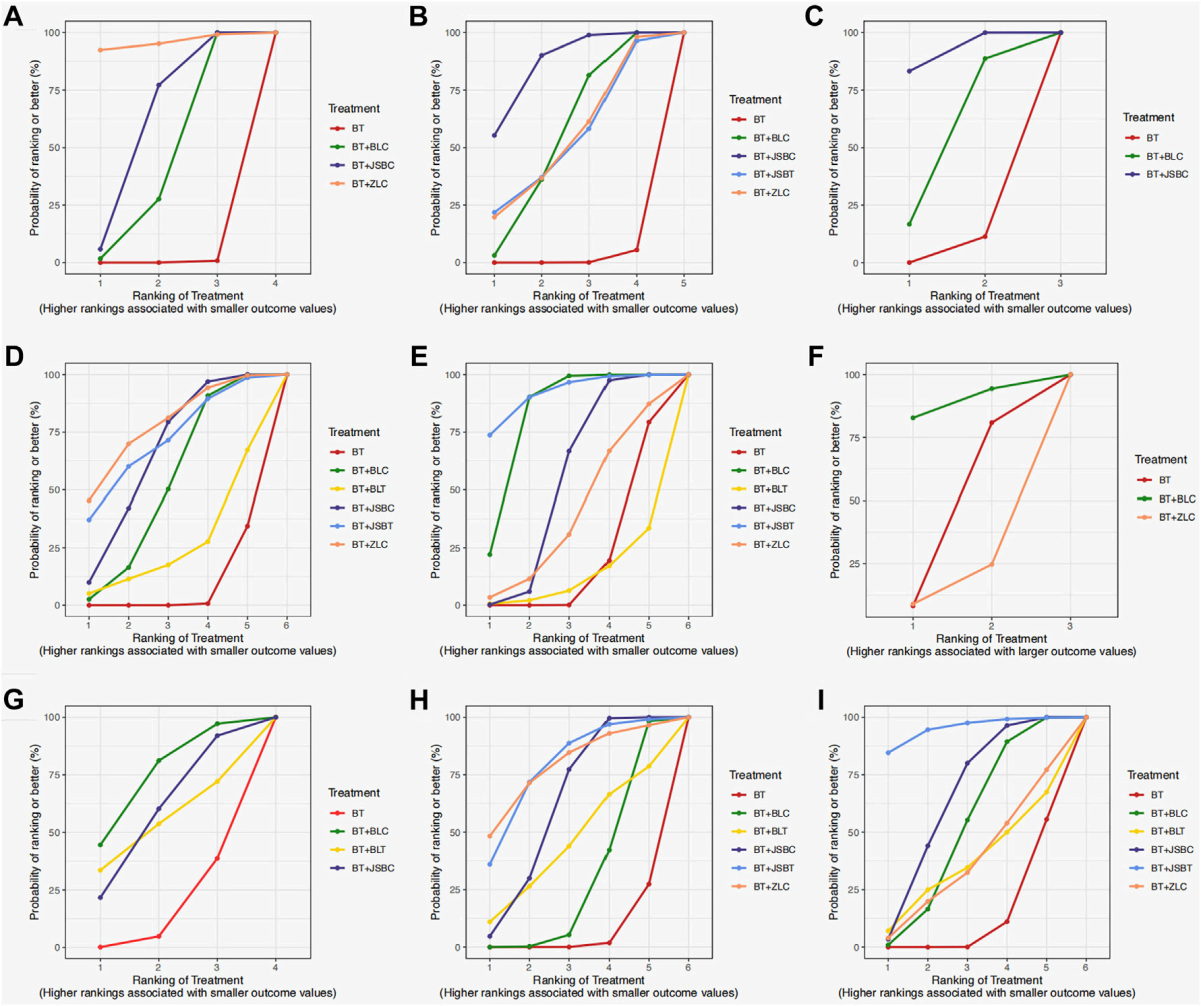
Surface under the cumulative ranking (SUCRA) plots on outcomes. Note: **(A)** 24-h urinary total protein; **(B)** urinary albumin excretion rate; **(C)** urinary albumin/creatinine ratio; **(D)** serum creatinine; **(E)** serum urea nitrogen; **(F)** estimated glomerular filtration rate; **(G)** adverse events; **(H)** fasting plasma glucose; and **(I)** glycated hemoglobin A1c. BT, basic treatment; BLC, Bailing capsule; BLT, Bailing tablet; JSBC, Jinshuibao capsule; JSBT, Jinshuibao tablet; and ZLC, Zhiling capsule.

Sixty-five RCTs compared the effectiveness of reducing 24-h UTP between the experimental and control groups, including 5,733 participants. Network evidence of 24-h UTP results showed that a total of four interventions were included (Figure 2A): BT and BT plus three *OS* preparations (BLC, JSBC, and ZLC). As the league table shows, the effectiveness of BT combined with any of the above three *OS* preparations on reducing urinary total protein was better than that of BT alone (Figure 3A). We drew the ranking probability plot and the SUCRA plot, from which we obtained the order of probability ranking results. The sequence from good to bad was as follows: BT + ZLC > BT + JSBC > BT + BLC > BT (Figure 4A).

#### 3.5.2 Important but not critical outcome: urinary albumin excretion rate

The UAER was reported in 83 trials including 5 interventions (BT and BT plus 4 *OS* preparations) and 6,617 patients. The four *OS* preparations were BLC, JSBC, JSBT, and ZLC (Figure 2B). The league table shows that the effectiveness of BT combined with any of the three *OS* preparations (BLC, JSBC, and ZLC) was superior to BT alone on reducing the UAER (Figure 3B). However, the combination of JSBT on reducing the UAER in DKD patients was not more significant than that of BT alone, and the difference was not statistically significant (MD 22.90, 95% CI −2.21 to 48.13, two trials). The SUCRA plot is sequenced as BT + JSBC, BT + BLC, BT + ZLC, BT + JSBT, and BT (Figure 4B).

#### 3.5.3 Important but not critical outcome: urinary albumin/creatinine ratio

A total of 10 RCTs, including 626 participants, were reported in the UACR. Network evidence of UACR results indicated that a total of three interventions were included (Figure 2C): BT and BT plus two *OS* preparations (BLC and JSBC). The league table showed that, when compared with BT, the MD [95% CI] for each intervention was as follows: BT + JSBC (−22.17 −[−31.23 to −14.09], eight trials) and BT + BLC (−12.53 [−32.83 to 11.40], two trials) (Figure 3C). According to the SUCRA values, “BT + JSBC” showed the highest effectiveness (SUCRA value of 91.58%), followed by “BT + BLC” (52.8%), while BT showed the lowest effectiveness (5.61%) (Figure 4C).

#### 3.5.4 Important but not critical outcome: serum creatinine

A total of 115 trials with 6 interventions and 9,802 individuals were included in the NMA of Scr. Network evidence of UACR results manifested that a total of six interventions were included: BT and BT plus all five *OS* preparations (Figure 2D). As the league table shows, the effectiveness of BT combined with any of the four *OS* preparations (BLC, JSBT, JSBC, or ZLC) was better than that of BT alone in reducing Scr levels (Figure 3D). However, the effectiveness of BT plus BLT in reducing Scr levels in DKD patients was not more significant than that of BT, and the difference was not statistically significant (MD 3.94, 95% CI −13.79 to 21.64, two trials). As the SUCRA plot shows, the sequence from good to bad was as follows: BT + ZLC > BT + JSBT > BT + JSBC > BT + BLC > BT + BLT > BT alone (Figure 4D).

#### 3.5.5 Important but not critical outcome: serum urea nitrogen

Eighty-one RCTs reported the effectiveness in reducing SUN levels, including 7,042 participants. Network evidence of SUN results indicated that a total of six interventions were included: BT and BT plus all five *OS* preparations (Figure 2E). The results of the NMA showed that the effectiveness of BT combined with BLC, JSBT, or JSBC was better than that of BT alone in reducing SUN levels. Nevertheless, the combination of BLT or ZLC did not have a superior effect in reducing SUN levels in DKD patients compared to BT alone (Figure 3E). The indirect comparison results suggested that “BT plus BLC” had a better effect than “BT plus JSBC” in decreasing SUN levels (MD −0.59, 95% CI −1.18 to −0.04); “BT combined with JSBT” was superior to “BT combined with BLT” (MD −2.35, 95% CI −4.53 to −0.19); and “BT plus BLC” was better than “BT plus BLT,” with an MD of −1.86 and a 95% CI (−3.60 to −0.14). The SUCRA plot is sequenced as BT + JSBT, BT + BLC, BT + JSBC, BT + ZLC, BT + BLT, and BT alone (Figure 4E).

#### 3.5.6 Important but not critical outcome: estimated glomerular filtration rate

A total of three trials with three interventions (BT, BT plus ZLC, and BLC) and 342 individuals were included in the NMA of the eGFR (Figure 2F). The league table showed no significant differences among the three interventions in improving the eGFR (Figure 3F). As the SUCRA plot shows, the sequence from good to bad was as follows: BT + BLC > BT alone > BT + ZLC (Figure 4F).

#### 3.5.7 Important but not critical outcome: adverse events

Twenty RCTs compared the AEs between groups. Network evidence of AE results showed that a total of four interventions were included: BT and BT plus JSBC, BLC, and BLT (Figure 2G). No serious AEs occurred in all included trials. The detailed adverse events are given in Supplementary Table S6. We analyzed the incidence of overall AEs between groups. As revealed in the league table, the results indicated that there was no significant statistical difference between the experimental group (co-intervention of BT and any of the three *OS* preparations) and the control group (BT alone) in the incidence of AEs (Figure 3G). On the basis of SUCRA values, the “BT + BLC” ranked the lowest in the incidence of an AE (SUCRA value of 74.23%), followed by “BT + JSBC” (57.63%) and “BT + BLT” (53.42%). BT was the highest in the incidence of an AE (14.72%) (Figure 4G).

#### 3.5.8 Other outcomes: fasting plasma glucose

FPG was reported in 53 studies including 6 interventions (BT and BT plus 5 *OS* preparations) and 4,549 patients (Figure 2H). BT combined with JSBC, JSBT, or BLC had better FPG reduction effectiveness than BT alone. However, the combination of ZLC or BLT has not been found. The indirect comparison results indicated that the effectiveness of BT combined with JSBC was higher than that of BT combined with BLC (MD −0.47, 95% CI −0.87 to −0.08) (Figure 3H). According to the SUCRA values, the “BT + ZLC” had the highest effectiveness (SUCRA value of 78.78%), followed by “BT + JSBT” (78.58%), “BT + JSBC” (62.36%), “BT + BLT” (45.13%), and “BT + BLC” (29.27%), while the BT had the lowest effectiveness (5.89%) (Figure 4H).

#### 3.5.9 Other outcomes: HbA1c

HbA1c was reported in 45 studies including 6 interventions (BT and BT plus 5 *OS* preparations) and 3,864 participants (Figure 2I). BT combined with JSBC, JSBT, or BLC had better HbA1c reduction effectiveness than BT alone. However, the combination of ZLC or BLT has not been found (Figure 3I). The SUCRA plot is sequenced as BT + JSBT, BT + JSBC, BT + BLC, BT + ZLC, BT + BLT, and BT alone (Figure 4I).

### 3.6 Sensitivity analyses

Seven trials did not report the course of *OS* preparations. We removed them and performed sensitivity analyses. We found that the addition of any of the *OS* capsular preparations was superior to RASi alone in reducing UAER levels. The remaining results of the direct comparisons were consistent with our main findings (Supplementary Figure S3). In addition, given that chronic diseases require long-term medication, we removed trials with treatment courses of 1 month or less, as well as trials without reported courses (18 trials). After removal, only the outcomes of Scr, SUN, and AEs were reported in the remaining trials that included the BLT intervention. The remaining results of the direct comparisons were consistent with the above sensitivity analysis results.

### 3.7 Publication bias

For the outcome measures of eGFR and UACR, since there were not enough (no more than 10 studies) included trials, no publication bias test was performed. The results of the counter-enhanced funnel plots combined with the trim and fill method showed that no significant publication bias appears to be found for the remaining outcome indicators (Supplementary Figure S2).

### 3.8 Grading of the evidence certainty

Combined evidence was not available because there was no closed loop. Overall, the evidence for nine outcomes was assessed. On the basis of GRADE assessment results, the overall certainty of direct evidence for main findings ranged from “very low” to “moderate,” and the certainty of indirect evidence ranged from “very low” to “low” (Supplementary Table S7). Among them, compared to RASi alone, the combination of JSBC and RASi showed beneficial effects in reducing 24-h UTP, UAER, UACR, Scr, SUN, FPG, and HbA1c levels, with moderate-certainty direct evidence. In addition, BLC plus RASi suggested significant effects in decreasing 24-h UTP, UAER, Scr, and SUN levels, with moderate-certainty direct evidence.

## 4 Discussion

### 4.1 Implications for healthcare and clinical practice

As a commonly used adjuvant medicine, *OS* preparations are used in the clinical practice of DKD treatment. Although the main medicinal ingredients of *OS* preparations on the market are isolated from wild *OS*, there may be differences in the therapeutic effects due to the differences in species name, fermentation techniques, and dosage forms. This is the first umbrella review and network meta-analysis generating the effectiveness and safety rankings of different *OS* preparations for DKD treatment, with a critical assessment of existing SRs. These results have brought beneficial implications for healthcare and clinical practice.

In this umbrella review, we specifically and comprehensively analyzed the effectiveness and safety of the combination of RASi and *OS* in treating DKD in the included 157 RCTs involving 13,130 patients. All RCTs included did not report end-point events, albuminuria progression, MACCEs, or patient-reported outcomes (PROs). For DKD, the prognosis is different in different stages of the disease. The cause–glomerular filtration rate–albuminuria (CGA) stage was proposed in the KDIGO Guidelines for the evaluation and management of CKD in 2012. Among them, G represents the eGFR degree, A represents the UACR degree, and the diagnostic stages are expressed by G1–5 and A1–3 (KDIGO, 2012). We found that Mogensen staging was used in most of the included RCTs, and some trials did not report disease staging. In addition, we know that Mogensen staging seems more appropriate for DKD caused by type 1 diabetes. Since the role of agents may not be the same in different stages of the disease, we need to select the most effective drugs at the specific stage of the intervention. This is something that needs to be improved in clinical practice and research in the future, with careful reporting of disease staging. Regarding the dose and duration of the intervention, this should be clearly reported in the RCT as the specifications vary from drug to drug. Unfortunately, the RCTs we included did not report well in this area. Therefore, we cannot analyze and explore the dose–response relationship between agents and diseases.

As is well known, urinary protein is a vital independent risk factor that affects the progression of DKD (Neuen et al., 2021). A Bayesian network meta-analysis revealed that the addition of any of the *OS* capsular preparations was superior to RASi alone in reducing 24-h UTP levels without additional adverse reactions. The “JSB capsule + RASi” therapy appeared to be beneficial in reducing urinary protein levels (including 24-h UTP, UAER, and UACR levels), protecting renal function (including Scr and SUN levels), and lowering blood glucose levels (including FPG and HbA1c levels) in patients with DKD. Although a few *OS* preparations did not have positive results in the outcomes of UAER, UACR, Scr, SUN, eGFR, FPG, and HbA1c levels, we found that the number of trials related to these outcomes was very rare, with almost only two trials. Therefore, the results need to be explained with caution as negative results may be related to a small number and insufficient statistical efficiency.

A recently published SR indicated that *OS* preparation combined with ACEIs/ARBs has a beneficial influence on renal function, proteinuria, and inflammation in DKD patients. However, no significant change in FPG and HbA1c levels was detected (Yan et al., 2023). Our results showed that RASi combined with JSBC, JSBT, or BLC had better FPG and HbA1c reduction effectiveness than BT alone. The reason may be that we used the Bayesian network meta-analysis method to analyze the effects of five different *OS* preparations on FPG and HbA1c levels. In contrast, only three *OS* preparations were included in the recent SR, and the number of studies and sample size corresponding to each *OS* preparation were lower than those included in our study. We found in some previous studies that *OS* also reduces insulin resistance and hypoglycemia *in vitro* and animal experiments (Kim et al., 2017; Liu et al., 2019). More clinical practice and studies are needed to confirm the effect of *OS* preparations on blood glucose levels in the future.

Based on the current results, *OS* preparations appear generally safe, and no serious adverse events were observed. Liver function impairment in individual patients in the experimental group was reported in three RCTs, one of which showed a doubling of aminotransferase levels, followed by a return to normal levels without stopping the intervention. The other two trials only reported improvement in liver function after symptomatic treatment, but no specific situation was reported. Renal function impairment in the experimental group was reported in four RCTs, one of which showed increased Scr levels but less than 30% of pre-medication levels. The research studies did not end the intervention and did not report subsequent prognosis. The other three trials only reported an improvement in kidney function after symptomatic treatment, but no detailed data on therapies and review results were reported. Because the subjects in the experimental group were taking *OS* and RASi at the same time, it is difficult to determine a causal relationship between the drugs. In addition, no follow-up information was recorded in the RCTs included, so long-term safety remains to be observed and determined in future healthcare and clinical practice.

Various factors are involved in the pathogenesis of DKD, including metabolic disorders, hemodynamic abnormalities, oxidative stress, inflammatory responses, immune abnormality, and epigenetics (Reddy et al., 2013; Ilyas et al., 2017; Tang and Yiu, 2020). RASi treat DKD mainly by improving hemodynamics (Reddy et al., 2013), and their inhibition of epigenetic changes and anti-inflammatory and antioxidant effects seems to be still being proven. *OS* can inhibit inflammation and improve antioxidation and immune regulation, thereby protecting the kidneys (Liu et al., 2022; Liu et al., 2022). Among the RCTs we included, there were trials that focused on the immune cells and inflammatory factors such as CD4/CD8 (5 trials), interleukin-6 (7 trials), and tumor necrosis factor-α (10 trials). The results all found that loading *OS* could significantly elevate the ratio of CD4/CD8 and reduce the levels of inflammation compared with basic treatment alone. We speculate that the combination of *OS* and RASi not only improves hemodynamics but also modulates immunity, anti-inflammatory, and antioxidant effects, which may be why the effect after adding *OS* based on the basic treatment is more significant. Of course, this is just the simplest hypothesis. Further in-depth studies are needed in future to elucidate the herb–drug interaction.

### 4.2 Future research recommendations to improve transparency and readability

In this study, the AMSTAR 2 tool was applied to appraise the methodological quality of the included SRs. Considering that the AMSTAR 2 scale was published in September 2017 (Shea et al., 2017), SRs published before 2017 may affect the quality inevitably due to the uncertainty of the evaluation criteria. Five of the SRs we included were published before 2017. We evaluated SRs published from 2013 to 2016 with “critically low” quality and SRs published from 2018 to 2023 with “high” and “critically low” quality. Regarding the critical items, the main problems focused on the non-reporting of research protocols, as well as literature exclusion lists. About the non-critical items, the problems centered on the unreported sources of funding and conflicts of interest. We used the RoB 2 tool to assess the methodological quality of the included RCTs. Because none of the trials had placebos, blinding of subjects and researchers could not be performed. The outcomes were almost all laboratory test indicators, which may be less affected by blinding. The current problem mainly focuses on the unclear reporting of randomized methods and selective reporting of outcomes. In addition, when it comes to patient-reported outcomes in future studies, the implementation of blinding is critical to the presentation of actual effects. Since the results may be affected by the subjective factors of participants and researchers, it may elevate the risk of detection bias and performance bias.

All of these problems and recommendations are intended to ensure scientific rigor and are essential to conducting high-quality research. In future studies, attention should be paid not only to the recommendations mentioned above to increase research transparency and methodological quality but also to present findings in accordance with international reporting standards and evaluate the preparations according to the core outcome set. This will make the results more readable and can be disseminated more widely for readers to obtain high-level research evidence.

### 4.3 Limitations

This review has several limitations. First, there were significant differences in the number and sample size of RCTs related to the five *OS* preparations. For example, the Zhiling capsule included only five trials and 421 participants, which may have an impact on the results. Second, the included RCTs did not report about allocation concealment, which could easily cause selective bias and potentially affect the accuracy and reliability of the analysis results. Third, all the subjects in this study were from China, and the generalizability of the results was limited. Fourth, there were no follow-up assessments conducted at the end of the intervention, which limits the ability to observe long-term effectiveness and safety. At last, only five trials of *OS* preparations for DKD treatment were retrieved. No evidence of other *OS* preparations for DKD was available, such as the Xinganbao capsule, Yong Chong Cao capsule, and Ningxinbao capsule. The evidence will need to be updated in the future.

## 5 Conclusion

In conclusion, there are some favorable effects in decreasing 24-h UTP levels in DKD patients using *OS* capsular preparations plus RASi. This study presents the effectiveness and safety ranking of *OS* preparations for DKD treatment. Our results need to be supplemented and improved by more high-quality and larger sample RCTs with a longer follow-up in the future. Future studies should focus more on the benefits of kidney disease progression and cardiovascular events. The herb–drug interaction should be explored in depth.

## Data Availability

The original contributions presented in the study are included in the article/Supplementary Material; further inquiries can be directed to the corresponding authors.
