# Supplementary material for: Ophiocordyceps sinensis preparations combined with the renin–angiotensin system inhibitor for diabetic kidney disease treatment: an umbrella review of systematic reviews and network meta-analysis
Source: Front Pharmacol. 2024 Apr 22;15:1360633. doi: 10.3389/fphar.2024.1360633 (PMC11075507; doi:10.3389/fphar.2024.1360633)
Supplement: Supplementary file 1 [file Table1.DOCX]

Supplementary Material

*Ophiocordyceps sinensis* preparations combined with renin-angiotensin system inhibitor for diabetic kidney disease: an umbrella review of systematic reviews and network meta-analysis

**Xue Xue^1^****^†^, Xin-yan Jin^2†^, Xing-lan Ye^3^, Ke-ying Li^3^, Jia-xuan Li^3^, Xue-han Liu^2^, Juan Bai^3^, Qiang Liu^4^, Bing-rui Zhang^5^, Xin-rong Zou^4^, Jun Yuan^6^, Chun-li Lu^7^, Fang-fang Zhao^8^, Jian-ping Liu^2^* and Xiao-qin Wang^4^***

*** Correspondence:**Jian-ping Liu: Liujp@bucm.edu.cn

Xiao-qin Wang: wangxiaoqin773@hotmail.com

# Supplementary Table 1A Searching strategies for electronic databases—Systematic reviews

| **Databases** | **Searching strategies** | **Results (n)** |
| --- | --- | --- |
| Chinese databases:  CNKI, SinoMed,  VIP, Wan Fang | #1.dongchongxiacao冬虫夏草 OR chongcaozhij虫草制剂 OR fajiaochongcaojunfen发酵虫草菌粉 OR bailing百令* OR jinshuibao金水宝* OR ningxinbao宁心宝* OR xinganbao心肝宝* OR yongchongcao蛹虫草* OR zhiling至灵*  #2.tangniaobingshenbing糖尿病肾病 OR tangniaobingshenzangbing糖尿病肾脏病 OR tangniaobingshenzangjibing糖尿病肾脏疾病 OR tangniaobingxingshenzangbing糖尿病性肾脏病 OR xiaokeshen消渴肾*  #3.xitongpingjia (系统评价) OR xitongzongshu (系统综述) OR Meta fenxi (meta分析) OR huicuifenxi (荟萃分析) OR xunzhengyanjiu (循证研究) OR yuanyanjiu (元研究) OR yuanfenxi (元分析)  #4.#1 AND #2 AND #3 | CNKI: 37  SinoMed: 29  VIP: 22  WanFang: 24 |
| PubMed | #1.ophiocordyceps sinensis[Title/Abstract] OR cordyceps sinensis[Title/Abstract] OR o. sinensis[Title/Abstract] OR c. sinensis[Title/Abstract] OR cordycepin[Title/Abstract] OR cordyceps militaris[Title/Abstract] OR winter worm summer herb[Title/Abstract] OR dongchongxiacao[Title/Abstract] OR dong chong xia cao[Title/Abstract] OR cordyceps preparation[Title/Abstract] OR bailing[Title/Abstract] OR bai ling[Title/Abstract] OR jinshuibao[Title/Abstract] OR jin shui bao[Title/Abstract] OR ningxinbao[Title/Abstract] OR ning xin bao[Title/Abstract] OR xinganbao[Title/Abstract] OR xin gan bao[Title/Abstract] OR yongchongcao[Title/Abstract] OR yong chong cao[Title/Abstract] OR zhiling[Title/Abstract] OR zhi ling[Title/Abstract]  #2.diabetic nephropath[Title/Abstract] OR nephropathies, diabetic[Title/Abstract] OR nephropathy, diabetic[Title/Abstract] OR diabetic kidney disease[Title/Abstract] OR diabetic kidney diseases[Title/Abstract] OR kidney disease, diabetic[Title/Abstract] OR kidney diseases, diabetic[Title/Abstract] OR DN[Title/Abstract] OR DKD[Title/Abstract] OR diabetic with chronic kidney disease[Title/Abstract] OR diabetic glomerulosclerosis[Title/Abstract] OR glomerulosclerosis, diabetic[Title/Abstract] OR diabetic glomerulosclerosis[Title/Abstract] OR intracapillary glomerulosclerosis[Title/Abstract] OR nodular glomerulosclerosis[Title/Abstract] OR glomerulosclerosis, nodular[Title/Abstract] OR kimmelstiel-wilson syndrome[Title/Abstract] OR kimmelstiel wilson syndrome[Title/Abstract] OR syndrome, kimmelstiel-wilson[Title/Abstract] OR kimmelstiel-wilson disease[Title/Abstract] OR kimmelstiel wilson disease[Title/Abstract]  #3.systematic review[Title/Abstract] OR meta analysis[Title/Abstract] OR meta-analysis[Title/Abstract] OR meta analyses[Title/Abstract] OR meta-analyses[Title/Abstract] OR meta synthesis[Title/Abstract] OR meta-synthesis[Title/Abstract] OR review[Title/Abstract]  #4.#1 AND #2 AND #3 | 10 |
| Web of Science | #1.SU=(ophiocordyceps sinensis OR cordyceps sinensis OR o. sinensis OR c. sinensis OR cordycepin OR cordyceps militaris OR winter worm summer herb OR dongchongxiacao OR dong chong xia cao OR cordyceps preparation OR bailing* OR bai ling* OR jinshuibao* OR jin shui bao* OR ningxinbao* OR ning xin bao* OR xinganbao* OR xin gan bao* OR yongchongcao* OR yong chong cao* OR zhiling* OR zhi ling*)  #2.SU=(diabetic nephropath* OR nephropathies, diabetic OR nephropathy, diabetic OR diabetic kidney disease OR diabetic kidney diseases OR kidney disease, diabetic OR kidney diseases, diabetic OR DN OR DKD OR diabetic with chronic kidney disease OR diabetic glomerulosclerosis OR glomerulosclerosis, diabetic OR diabetic glomerulosclerosis OR intracapillary glomerulosclerosis OR nodular glomerulosclerosis OR glomerulosclerosis, nodular OR kimmelstiel-wilson syndrome OR kimmelstiel wilson syndrome OR syndrome, kimmelstiel-wilson OR kimmelstiel-wilson disease OR kimmelstiel wilson disease )  #3.SU=(systematic review OR meta analysis OR meta-analysis OR meta analyses OR meta-analyses OR meta synthesis OR meta-synthesis OR review)  #4.#1 AND #2 AND #3 | 13 |
| The Cochrane library | #1.ophiocordyceps sinensis OR cordyceps sinensis OR o. sinensis OR c. sinensis OR cordycepin OR cordyceps militaris OR winter worm summer herb OR dongchongxiacao OR dong chong xia cao OR cordyceps preparation OR bailing* OR bai ling* OR jinshuibao* OR jin shui bao* OR ningxinbao* OR ning xin bao* OR xinganbao* OR xin gan bao* OR yongchongcao* OR yong chong cao* OR zhiling* OR zhi ling* in Title Abstract Keyword  #2.diabetic nephropath* OR nephropathies, diabetic OR nephropathy, diabetic OR diabetic kidney disease OR diabetic kidney diseases OR kidney disease, diabetic OR kidney diseases, diabetic OR DN OR DKD OR diabetic with chronic kidney disease OR diabetic glomerulosclerosis OR glomerulosclerosis, diabetic OR diabetic glomerulosclerosis OR intracapillary glomerulosclerosis OR nodular glomerulosclerosis OR glomerulosclerosis, nodular OR kimmelstiel-wilson syndrome OR kimmelstiel wilson syndrome OR syndrome, kimmelstiel-wilson OR kimmelstiel-wilson disease OR kimmelstiel wilson disease in Title Abstract Keyword  #3.systematic review OR meta analysis OR meta-analysis OR meta analyses OR meta-analyses OR meta synthesis OR meta-synthesis OR review in Title Abstract Keyword  #4.#1 AND #2 AND #3 | 0 |
| EMBASE | #1.'ophiocordyceps sinensis':ti,ab,kw OR 'cordyceps sinensis':ti,ab,kw OR 'o. sinensis':ti,ab,kw OR 'c. sinensis':ti,ab,kw OR cordycepin:ti,ab,kw OR 'cordyceps militaris':ti,ab,kw OR 'winter worm summer herb':ti,ab,kw OR dongchongxiacao:ti,ab,kw OR 'dong chong xia cao':ti,ab,kw OR 'cordyceps preparation':ti,ab,kw OR bailing*:ti,ab,kw OR 'bai ling*':ti,ab,kw OR jinshuibao*:ti,ab,kw OR 'jin shui bao*':ti,ab,kw OR ningxinbao*:ti,ab,kw OR 'ning xin bao*':ti,ab,kw OR xinganbao*:ti,ab,kw OR 'xin gan bao*':ti,ab,kw OR yongchongcao*:ti,ab,kw OR 'yong chong cao*':ti,ab,kw OR zhiling*:ti,ab,kw OR 'zhi ling*':ti,ab,kw  #2.'diabetic nephropath*':ti,ab,kw OR 'nephropathies, diabetic':ti,ab,kw OR 'nephropathy, diabetic':ti,ab,kw OR 'diabetic kidney disease':ti,ab,kw OR 'diabetic kidney diseases':ti,ab,kw OR 'kidney disease, diabetic':ti,ab,kw OR 'kidney diseases, diabetic':ti,ab,kw OR dn:ti,ab,kw OR dkd:ti,ab,kw OR 'diabetic with chronic kidney disease':ti,ab,kw OR 'glomerulosclerosis, diabetic':ti,ab,kw OR 'diabetic glomerulosclerosis':ti,ab,kw OR 'intracapillary glomerulosclerosis':ti,ab,kw OR 'nodular glomerulosclerosis':ti,ab,kw OR 'glomerulosclerosis, nodular':ti,ab,kw OR 'kimmelstiel-wilson syndrome':ti,ab,kw OR 'kimmelstiel wilson syndrome':ti,ab,kw OR 'syndrome, kimmelstiel-wilson':ti,ab,kw OR 'kimmelstiel-wilson disease':ti,ab,kw OR 'kimmelstiel wilson disease':ti,ab,kw  #3.'systematic review':ti,ab,kw OR 'meta analysis':ti,ab,kw OR 'meta analyses':ti,ab,kw OR 'meta synthesis':ti,ab,kw OR review:ti,ab,kw  #4.#1 AND #2 AND #3 | 13 |

# Supplementary Table 1B Searching strategies for electronic databases—Randomized controlled trials

| **Databases** | **Searching strategies** | **Results (n)** |
| --- | --- | --- |
| Chinese databases:  CNKI, SinoMed,  VIP, Wan Fang | #1.dongchongxiacao冬虫夏草 OR chongcaozhij虫草制剂 OR fajiaochongcaojunfen发酵虫草菌粉 OR bailing百令* OR jinshuibao金水宝* OR ningxinbao宁心宝* OR xinganbao心肝宝* OR yongchongcao蛹虫草* OR zhiling至灵*  #2.tangniaobingshenbing糖尿病肾病 OR tangniaobingshenzangbing糖尿病肾脏病 OR tangniaobingshenzangjibing糖尿病肾脏疾病 OR tangniaobingxingshenzangbing糖尿病性肾脏病 OR xiaokeshen消渴肾*  #3.RCT OR randomize dontrolled trial (随机对照试验) OR randomized controlled study (随机对照研究) OR clinical trial (临床试验) OR clinical study(临床研究)  #4.Date: 2020/08/10 - 2023/05/15  #5.#1 AND #2 AND #3 AND #4 | CNKI: 49  SinoMed: 26  VIP: 47  WanFang: 65 |
| PubMed | #1.ophiocordyceps sinensis[Title/Abstract] OR cordyceps sinensis[Title/Abstract] OR o. sinensis[Title/Abstract] OR c. sinensis[Title/Abstract] OR cordycepin[Title/Abstract] OR cordyceps militaris[Title/Abstract] OR winter worm summer herb[Title/Abstract] OR dongchongxiacao[Title/Abstract] OR dong chong xia cao[Title/Abstract] OR cordyceps preparation[Title/Abstract] OR bailing[Title/Abstract] OR bai ling[Title/Abstract] OR jinshuibao[Title/Abstract] OR jin shui bao[Title/Abstract] OR ningxinbao[Title/Abstract] OR ning xin bao[Title/Abstract] OR xinganbao[Title/Abstract] OR xin gan bao[Title/Abstract] OR yongchongcao[Title/Abstract] OR yong chong cao[Title/Abstract] OR zhiling[Title/Abstract] OR zhi ling[Title/Abstract]  #2.diabetic nephropath[Title/Abstract] OR nephropathies, diabetic[Title/Abstract] OR nephropathy, diabetic[Title/Abstract] OR diabetic kidney disease[Title/Abstract] OR diabetic kidney diseases[Title/Abstract] OR kidney disease, diabetic[Title/Abstract] OR kidney diseases, diabetic[Title/Abstract] OR DN[Title/Abstract] OR DKD[Title/Abstract] OR diabetic with chronic kidney disease[Title/Abstract] OR diabetic glomerulosclerosis[Title/Abstract] OR glomerulosclerosis, diabetic[Title/Abstract] OR diabetic glomerulosclerosis[Title/Abstract] OR intracapillary glomerulosclerosis[Title/Abstract] OR nodular glomerulosclerosis[Title/Abstract] OR glomerulosclerosis, nodular[Title/Abstract] OR kimmelstiel-wilson syndrome[Title/Abstract] OR kimmelstiel wilson syndrome[Title/Abstract] OR syndrome, kimmelstiel-wilson[Title/Abstract] OR kimmelstiel-wilson disease[Title/Abstract] OR kimmelstiel wilson disease[Title/Abstract]  #3.RCT[Title/Abstract] OR randomize dontrolled tria[Title/Abstract] OR randomized controlled study[Title/Abstract] OR clinical trial[Title/Abstract] OR clinical study[Title/Abstract]  #4.Year: 2020-2023  #5.#1 AND #2 AND #3 AND #4 | 0 |
| Web of Science | #1.TS=(ophiocordyceps sinensis OR cordyceps sinensis OR o. sinensis OR c. sinensis OR cordycepin OR cordyceps militaris OR winter worm summer herb OR dongchongxiacao OR dong chong xia cao OR cordyceps preparation OR bailing* OR bai ling* OR jinshuibao* OR jin shui bao* OR ningxinbao* OR ning xin bao* OR xinganbao* OR xin gan bao* OR yongchongcao* OR yong chong cao* OR zhiling* OR zhi ling*)  #2.TS=(diabetic nephropath* OR nephropathies, diabetic OR nephropathy, diabetic OR diabetic kidney disease OR diabetic kidney diseases OR kidney disease, diabetic OR kidney diseases, diabetic OR DN OR DKD OR diabetic with chronic kidney disease OR diabetic glomerulosclerosis OR glomerulosclerosis, diabetic OR diabetic glomerulosclerosis OR intracapillary glomerulosclerosis OR nodular glomerulosclerosis OR glomerulosclerosis, nodular OR kimmelstiel-wilson syndrome OR kimmelstiel wilson syndrome OR syndrome, kimmelstiel-wilson OR kimmelstiel-wilson disease OR kimmelstiel wilson disease)  #3.TS=(RCT OR randomize dontrolled trial OR randomized controlled study OR clinical trial OR clinical study)  #4.Date: 2020-08-10 to 2023-05-15  #5.#1 AND #2 AND #3 AND #4 | 10 |
| The Cochrane library | #1.ophiocordyceps sinensis OR cordyceps sinensis OR o. sinensis OR c. sinensis OR cordycepin OR cordyceps militaris OR winter worm summer herb OR dongchongxiacao OR dong chong xia cao OR cordyceps preparation OR bailing* OR bai ling* OR jinshuibao* OR jin shui bao* OR ningxinbao* OR ning xin bao* OR xinganbao* OR xin gan bao* OR yongchongcao* OR yong chong cao* OR zhiling* OR zhi ling* in Title Abstract Keyword  #2.diabetic nephropath* OR nephropathies, diabetic OR nephropathy, diabetic OR diabetic kidney disease OR diabetic kidney diseases OR kidney disease, diabetic OR kidney diseases, diabetic OR DN OR DKD OR diabetic with chronic kidney disease OR diabetic glomerulosclerosis OR glomerulosclerosis, diabetic OR diabetic glomerulosclerosis OR intracapillary glomerulosclerosis OR nodular glomerulosclerosis OR glomerulosclerosis, nodular OR kimmelstiel-wilson syndrome OR kimmelstiel wilson syndrome OR syndrome, kimmelstiel-wilson OR kimmelstiel-wilson disease OR kimmelstiel wilson disease in Title Abstract Keyword  #3.RCT OR randomize dontrolled trial OR randomized controlled study OR clinical trial OR clinical study in Title Abstract Keyword  #4.Date Between Oct 2020 and May 2023  #5.#1 AND #2 AND #3 AND #4 | 3 |
| EMBASE | #1.'ophiocordyceps sinensis':ti,ab,kw OR 'cordyceps sinensis':ti,ab,kw OR 'o. sinensis':ti,ab,kw OR 'c. sinensis':ti,ab,kw OR cordycepin:ti,ab,kw OR 'cordyceps militaris':ti,ab,kw OR 'winter worm summer herb':ti,ab,kw OR dongchongxiacao:ti,ab,kw OR 'dong chong xia cao':ti,ab,kw OR 'cordyceps preparation':ti,ab,kw OR bailing*:ti,ab,kw OR 'bai ling*':ti,ab,kw OR jinshuibao*:ti,ab,kw OR 'jin shui bao*':ti,ab,kw OR ningxinbao*:ti,ab,kw OR 'ning xin bao*':ti,ab,kw OR xinganbao*:ti,ab,kw OR 'xin gan bao*':ti,ab,kw OR yongchongcao*:ti,ab,kw OR 'yong chong cao*':ti,ab,kw OR zhiling*:ti,ab,kw OR 'zhi ling*':ti,ab,kw  #2.'diabetic nephropath*':ti,ab,kw OR 'nephropathies, diabetic':ti,ab,kw OR 'nephropathy, diabetic':ti,ab,kw OR 'diabetic kidney disease':ti,ab,kw OR 'diabetic kidney diseases':ti,ab,kw OR 'kidney disease, diabetic':ti,ab,kw OR 'kidney diseases, diabetic':ti,ab,kw OR dn:ti,ab,kw OR dkd:ti,ab,kw OR 'diabetic with chronic kidney disease':ti,ab,kw OR 'glomerulosclerosis, diabetic':ti,ab,kw OR 'diabetic glomerulosclerosis':ti,ab,kw OR 'intracapillary glomerulosclerosis':ti,ab,kw OR 'nodular glomerulosclerosis':ti,ab,kw OR 'glomerulosclerosis, nodular':ti,ab,kw OR 'kimmelstiel-wilson syndrome':ti,ab,kw OR 'kimmelstiel wilson syndrome':ti,ab,kw OR 'syndrome, kimmelstiel-wilson':ti,ab,kw OR 'kimmelstiel-wilson disease':ti,ab,kw OR 'kimmelstiel wilson disease':ti,ab,kw  #3.'RCT':ti,ab,kw OR 'randomize dontrolled trial':ti,ab,kw OR 'randomized controlled study':ti,ab,kw OR 'clinical trial':ti,ab,kw OR 'clinical study':ti,ab,kw  #4.[2020-2023]/py  #5.#1 AND #2 AND #3 AND #4 | 2 |
