# Supplementary material for: Ophiocordyceps sinensis preparations combined with the renin–angiotensin system inhibitor for diabetic kidney disease treatment: an umbrella review of systematic reviews and network meta-analysis
Source: Front Pharmacol. 2024 Apr 22;15:1360633. doi: 10.3389/fphar.2024.1360633 (PMC11075507; doi:10.3389/fphar.2024.1360633)
Supplement: Supplementary file 3 [file Table7.DOCX]

Supplementary Material

*Ophiocordyceps sinensis* preparations combined with renin-angiotensin system inhibitor for diabetic kidney disease: an umbrella review of systematic reviews and network meta-analysis

**Xue Xue^1^****^†^, Xin-yan Jin^2†^, Xing-lan Ye^3^, Ke-ying Li^3^, Jia-xuan Li^3^, Xue-han Liu^2^, Juan Bai^3^, Qiang Liu^4^, Bing-rui Zhang^5^, Xin-rong Zou^4^, Jun Yuan^6^, Chun-li Lu^7^, Fang-fang Zhao^8^, Jian-ping Liu^2^* and Xiao-qin Wang^4^***

*** Correspondence:**Jian-ping Liu: Liujp@bucm.edu.cn

Xiao-qin Wang: wangxiaoqin773@hotmail.com

# Supplementary Table 7 Certainty of evidence for primary findings

| Outcomes | Comparison | No. of Studies (Participants) for direct pairwise | Direct Evidence | | | Indirect Evidence | | |
| --- | --- | --- | --- | --- | --- | --- | --- | --- |
|  |  |  | MD (95% CI) | Quality | Reason(s) for downgrading | MD (95% CI) | Quality | Reason(s) for downgrading |
| 24h UTP | BT+BLC vs. BT | 32(2740) | 228.88(133.59,328.27) | ⊕⊕⊕○moderate | a | — | — | — |
|  | BT+JSBC vs. BT | 32(2895) | 276.95(181.88,375.35) | ⊕⊕⊕○moderate | a | — | — | — |
|  | BT+ZLC vs. BT | 1(98) | 711.14(136.00,1286.83) | ⊕○○○very low | ac | — | — | — |
|  | BT+BLC vs. BT+JSBC | 32(2740) vs. 32(2895) | — | — | — | 48.02(-88.26,184.26) | ⊕⊕○○low | ab |
|  | BT+BLC vs. BT+ZLC | 32(2740) vs. 1(98) | — | — | — | 482.00(-103.44,1064.26) | ⊕⊕○○low | ab |
|  | BT+JSBC vs. BT+ZLC | 32(2895) vs. 1(98) | — | — | — | 434.00(-150.92,1016.54) | ⊕⊕○○low | ab |
| UAER | BT+BLC vs. BT | 47(3761) | 25.21(19.87,30.55) | ⊕⊕⊕○moderate | a | — | — | — |
|  | BT+JSBC vs. BT | 31(2481) | 31.64(24.98,38.44) | ⊕⊕⊕○moderate | a | — | — | — |
|  | BT+JSBT vs. BT | 2(174) | 22.90(-2.21,48.13) | ⊕○○○very low | ac | — | — | — |
|  | BT+ZLC vs. BT | 3(201) | 23.26(1.20,45.40) | ⊕⊕○○low | ab | — | — | — |
|  | BT+BLC vs. BT+JSBC | 47(3761) vs. 31(2481) | — | — | — | 6.43(-2.11,15.07) | ⊕⊕○○low | ab |
|  | BT+BLC vs. BT+JSBT | 47(3761) vs. 2(174) | — | — | — | 2.30(-23.49,28.00) | ⊕⊕○○low | ab |
|  | BT+BLC vs. BT+ZLC | 47(3761) vs. 3(201) | — | — | — | 1.94(-20.84,24.68) | ⊕⊕○○low | ab |
|  | BT+JSBC vs. BT+JSBT | 31(2481) vs. 2(174) | — | — | — | 8.76(-17.30,34.81) | ⊕⊕○○low | ab |
|  | BT+JSBC vs. BT+ZLC | 31(2481) vs. 3(201) | — | — | — | 8.39(-14.70,31.48) | ⊕⊕○○low | ab |
|  | BT+JSBT vs. BT+ZLC | 2(174) vs. 3(201) | — | — | — | 0.40(-33.11,33.86) | ⊕⊕○○low | ab |
| UACR | BT+BLC vs. BT | 2(122) | 12.53(-11.40,32.83) | ⊕○○○very low | ac | — | — | — |
|  | BT+JSBC vs. BT | 8(504) | 22.17(14.09,31.23) | ⊕⊕⊕○moderate | a | — | — | — |
|  | BT+BLC vs. BT+JSBC | 2(122) vs. 8(504) | — | — | — | 9.65(-11.79,35.68) | ⊕○○○very low | ac |
| Scr | BT+BLC vs. BT | 59(4918) | 11.85(8.49,15.20) | ⊕⊕⊕○moderate | a | — | — | — |
|  | BT+BLT vs. BT | 2(178) | 3.94(-13.79,21.64) | ⊕○○○very low | ac | — | — | — |
|  | BT+JSBC vs. BT | 46(4033) | 13.50(9.62,17.38) | ⊕⊕⊕○moderate | a | — | — | — |
|  | BT+JSBT vs. BT | 3(252) | 15.58(1.11,30.08) | ⊕⊕○○low | ab | — | — | — |
|  | BT+ZLC vs. BT | 5(421) | 16.93(4.35,29.54) | ⊕⊕○○low | ab | — | — | — |
|  | BT+BLC vs. BT+BLT | 59(4918) vs. 2(178) | — | — | — | 7.90(-10.11,25.97) | ⊕⊕○○low | ab |
|  | BT+BLC vs. BT+JSBC | 59(4918)vs. 46(4033) | — | — | — | 1.65(-3.48,6.78) | ⊕⊕○○low | ab |
|  | BT+BLC vs. BT+JSBT | 59(4918) vs. 3(252) | — | — | — | 3.74(-11.14,18.64) | ⊕⊕○○low | ab |
|  | BT+BLC vs. BT+ZLC | 59(4918) vs. 5(421) | — | — | — | 5.08(-7.95,18.12) | ⊕⊕○○low | ab |
|  | BT+BLT vs. BT+JSBC | 2(178) vs. 46(4033) | — | — | — | 9.55(-8.56,27.70) | ⊕⊕○○low | ab |
|  | BT+BLT vs. BT+JSBT | 2(178) vs. 3(252) | — | — | — | 11.65(-11.27,34.53) | ⊕⊕○○low | ab |
|  | BT+BLT vs. BT+ZLC | 2(178) vs. 5(421) | — | — | — | 12.98(-8.71,34.70) | ⊕⊕○○low | ab |
|  | BT+JSBC vs. BT+JSBT | 46(4033) vs. 3(252) | — | — | — | 2.09(-12.91,17.10) | ⊕⊕○○low | ab |
|  | BT+JSBC vs. BT+ZLC | 46(4033) vs. 5(421) | — | — | — | 3.42(-9.74,16.62) | ⊕⊕○○low | ab |
|  | BT+JSBT vs. BT+ZLC | 3(252) vs. 5(421) | — | — | — | 1.33(-17.82,20.55) | ⊕⊕○○low | ab |
| SUN | BT+BLC vs. BT | 39(3445) | 1.38(1.00,1.79) | ⊕⊕⊕○moderate | a | — | — | — |
|  | BT+BLT vs. BT | 2(178) | 0.48(-1.20,2.16) | ⊕○○○very low | ac | — | — | — |
|  | BT+JSBC vs. BT | 34(2966) | 0.79(0.38,1.20) | ⊕⊕⊕○moderate | a | — | — | — |
|  | BT+JSBT vs. BT | 3(252) | 1.87(0.50,3.25) | ⊕⊕○○low | ab | — | — | — |
|  | BT+ZLC vs. BT | 3(201) | 0.42(-0.96,1.81) | ⊕⊕○○low | ab | — | — | — |
|  | BT+BLC vs. BT+BLT | 39(3445) vs. 2(178) | — | — | — | 1.86(0.14,3.60) | ⊕⊕○○low | ab |
|  | BT+BLC vs. BT+JSBC | 39(3445) vs. 34(2966) | — | — | — | 0.59(0.04,1.18) | ⊕⊕○○low | ab |
|  | BT+BLC vs. BT+JSBT | 39(3445) vs. 3(252) | — | — | — | 0.49(-0.95,1.91) | ⊕⊕○○low | ab |
|  | BT+BLC vs. BT+ZLC | 39(3445) vs. 3(201) | — | — | — | 0.96(-0.47,2.41) | ⊕⊕○○low | ab |
|  | BT+BLT vs. BT+JSBC | 2(178) vs. 34(2966) | — | — | — | 1.26(-0.47,3.00) | ⊕⊕○○low | ab |
|  | BT+BLT vs. BT+JSBT | 2(178) vs. 3(252) | — | — | — | 2.35(0.19,44.53) | ⊕⊕○○low | ab |
|  | BT+BLT vs. BT+ZLC | 2(178) vs. 3(201) | — | — | — | 0.90(-1.27,3.08) | ⊕⊕○○low | ab |
|  | BT+JSBC vs. BT+JSBT | 34(2966) vs. 3(252) | — | — | — | 1.09(-0.34,2.52) | ⊕⊕○○low | ab |
|  | BT+JSBC vs. BT+ZLC | 34(2966) vs. 3(201) | — | — | — | 0.36(-1.07,1.81) | ⊕⊕○○low | ab |
|  | BT+JSBT vs. BT+ZLC | 3(252) vs. 3(201) | — | — | — | 1.45(-0.50,3.40) | ⊕⊕○○low | ab |
| eGFR | BT+BLC vs. BT | 2(220) | 5.24(-4.30,12.61) | ⊕⊕○○low | ab | — | — | — |
|  | BT+ZLC vs. BT | 1(122) | 2.99(-7.70,13.66) | ⊕○○○very low | ac | — | — | — |
|  | BT+BLC vs. BT+ZLC | 2(220) vs. 1(122) | — | — | — | 8.35(-6.53,20.68) | ⊕⊕○○low | ab |
| AEs | BT+BLC vs. BT | 11(868) | 1.60(0.94,2.86) | ⊕⊕○○low | ab | — | — | — |
|  | BT+BLT vs. BT | 1(80) | 1.35(0.40,4.87) | ⊕○○○very low | ac | — | — | — |
|  | BT+JSBC vs. BT | 8(802) | 1.37(0.81,2.41) | ⊕⊕⊕○moderate | a | — | — | — |
|  | BT+BLC vs. BT+BLT | 11(868) vs. 1(80) | — | — | — | 1.18(0.30,4.63) | ⊕⊕○○low | ab |
|  | BT+BLC vs. BT+JSBC | 11(868) vs. 8(802) | — | — | — | 1.17(0.54,2.54) | ⊕⊕○○low | ab |
|  | BT+BLT vs. BT+JSBC | 1(80) vs. 8(802) | — | — | — | 1.01(0.26,3.93) | ⊕⊕○○low | ab |
| FPG | BT+BLC vs. BT | 32(2620) | 0.24(0.00,0.48) | ⊕⊕○○low | ab | — | — | — |
|  | BT+BLT vs. BT | 1(98) | 0.49(-0.78,1.76) | ⊕○○○very low | ac | — | — | — |
|  | BT+JSBC vs. BT | 17(1573) | 0.71(0.40,1.03) | ⊕⊕⊕○moderate | a | — | — | — |
|  | BT+JSBT vs. BT | 2(158) | 1.06(0.15,1.96) | ⊕○○○very low | ac | — | — | — |
|  | BT+ZLC vs. BT | 1(100) | 1.16(-0.15,2.47) | ⊕○○○very low | ac | — | — | — |
|  | BT+BLC vs. BT+BLT | 32(2620) vs. 1(98) | — | — | — | 0.25(-1.04,1.54) | ⊕⊕○○low | ab |
|  | BT+BLC vs. BT+JSBC | 32(2620) vs. 17(1573) | — | — | — | 0.47(0.08,0.87) | ⊕⊕○○low | ab |
|  | BT+BLC vs. BT+JSBT | 32(2620) vs. 2(158) | — | — | — | 0.82(-0.12,1.75) | ⊕⊕○○low | ab |
|  | BT+BLC vs. BT+ZLC | 32(2620) vs. 1(100) | — | — | — | 0.92(-0.41,2.25) | ⊕⊕○○low | ab |
|  | BT+BLT vs. BT+JSBC | 1(98) vs. 17(1573) | — | — | — | 0.22(-1.08,1.53) | ⊕⊕○○low | ab |
|  | BT+BLT vs. BT+JSBT | 1(98) vs. 2(158) | — | — | — | 0.57(-0.99,2.12) | ⊕⊕○○low | ab |
|  | BT+BLT vs. BT+ZLC | 1(98) vs. 1(100) | — | — | — | 0.67(-1.15,2.48) | ⊕⊕○○low | ab |
|  | BT+JSBC vs. BT+JSBT | 17(1573) vs. 2(158) | — | — | — | 0.34(-0.62,1.30) | ⊕⊕○○low | ab |
|  | BT+JSBC vs. BT+ZLC | 17(1573) vs. 1(100) | — | — | — | 0.44(-0.90,1.79) | ⊕⊕○○low | ab |
|  | BT+JSBT vs. BT+ZLC | 2(158) vs. 1(100) | — | — | — | 0.10(-1.49,1.69) | ⊕⊕○○low | ab |
| HbA1c | BT+BLC vs. BT | 21(1798) | 0.40(0.15,0.66) | ⊕⊕○○low | ab | — | — | — |
|  | BT+BLT vs. BT | 1(98) | 0.19(-0.94,1.32) | ⊕○○○very low | ac | — | — | — |
|  | BT+JSBC vs. BT | 19(1612) | 0.51(0.24,0.78) | ⊕⊕⊕○moderate | a | — | — | — |
|  | BT+JSBT vs. BT | 2(158) | 1.16(0.36,1.96) | ⊕○○○very low | ac | — | — | — |
|  | BT+ZLC vs. BT | 2(198) | 0.22(-0.61,1.06) | ⊕○○○very low | ac | — | — | — |
|  | BT+BLC vs. BT+BLT | 21(1798) vs. 1(98) | — | — | — | 0.21(-0.95,1.38) | ⊕⊕○○low | ab |
|  | BT+BLC vs. BT+JSBC | 21(1798) vs. 19(1612) | — | — | — | 0.11(-0.26,0.48) | ⊕⊕○○low | ab |
|  | BT+BLC vs. BT+JSBT | 21(1798) vs. 2(158) | — | — | — | 0.75(-0.08,1.59) | ⊕⊕○○low | ab |
|  | BT+BLC vs. BT+ZLC | 21(1798) vs. 2(198) | — | — | — | 0.18(-0.70,1.05) | ⊕⊕○○low | ab |
|  | BT+BLT vs. BT+JSBC | 1(98) vs. 19(1612) | — | — | — | 0.32(-0.84,1.49) | ⊕⊕○○low | ab |
|  | BT+BLT vs. BT+JSBT | 1(98) vs. 2(158) | — | — | — | 0.97(-0.42,2.36) | ⊕⊕○○low | ab |
|  | BT+BLT vs. BT+ZLC | 1(98) vs. 2(198) | — | — | — | 0.03(-1.37,1.45) | ⊕⊕○○low | ab |
|  | BT+JSBC vs. BT+JSBT | 19(1612) vs. 2(158) | — | — | — | 0.65(-0.20,1.48) | ⊕⊕○○low | ab |
|  | BT+JSBC vs. BT+ZLC | 19(1612) vs. 2(198) | — | — | — | 0.29(-0.59,1.16) | ⊕⊕○○low | ab |
|  | BT+JSBT vs. BT+ZLC | 2(158) vs. 2(198) | — | — | — | 0.93(-0.23,2.09) | ⊕⊕○○low | ab |

**Note:** a. Downgraded one level for high risk of bias. b. Downgraded one level for imprecision since 95% CI is wide and/or crosses unity. c. Downgraded two levels for imprecision since 95% CI is very wide and crosses unity.
