# Supplementary material for: Ophiocordyceps sinensis preparations combined with the renin–angiotensin system inhibitor for diabetic kidney disease treatment: an umbrella review of systematic reviews and network meta-analysis
Source: Front Pharmacol. 2024 Apr 22;15:1360633. doi: 10.3389/fphar.2024.1360633 (PMC11075507; doi:10.3389/fphar.2024.1360633)
Supplement: Supplementary file 5 [file Image2.PDF]

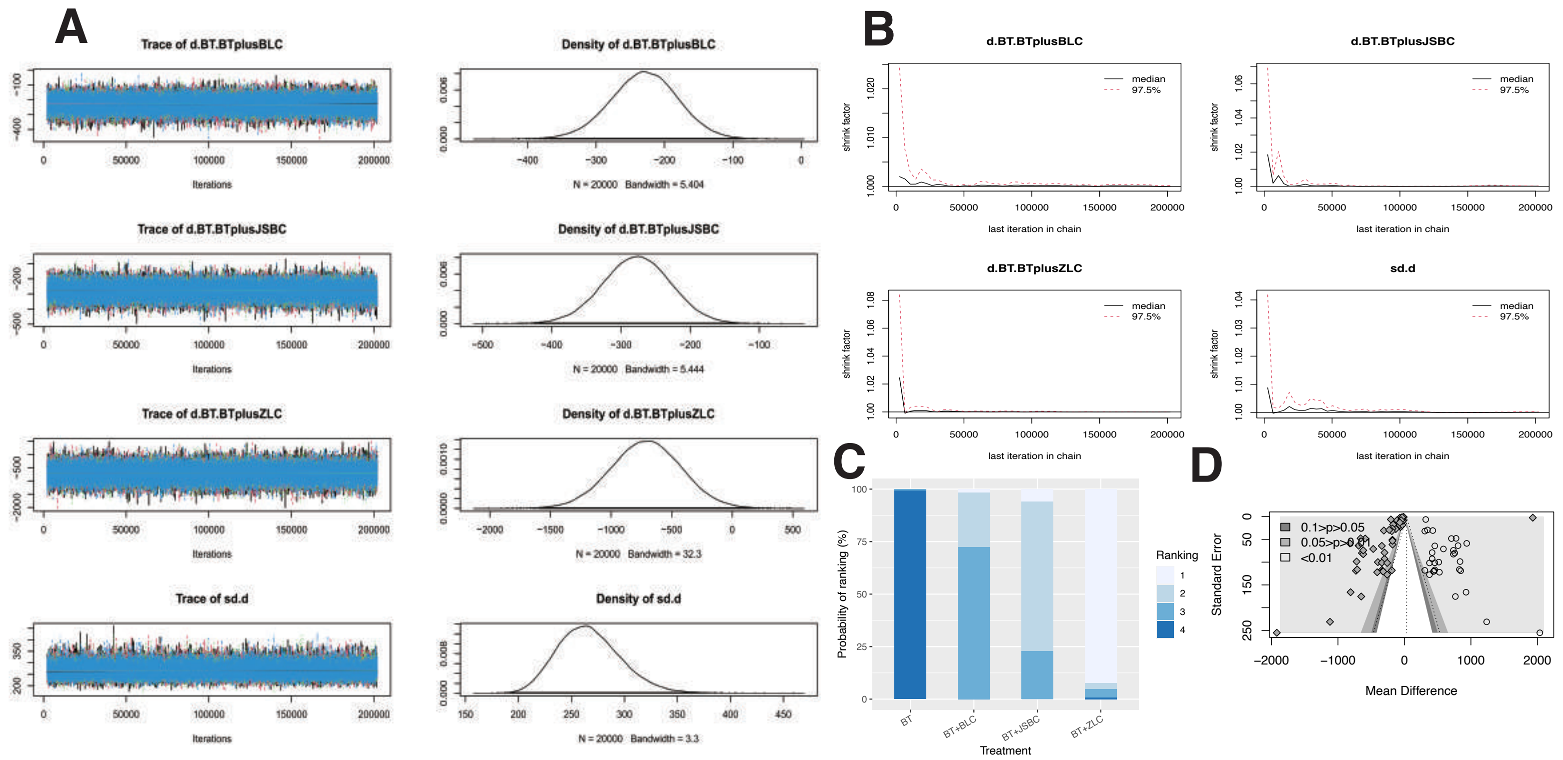

eFig.2a 24 hours urinary total protein

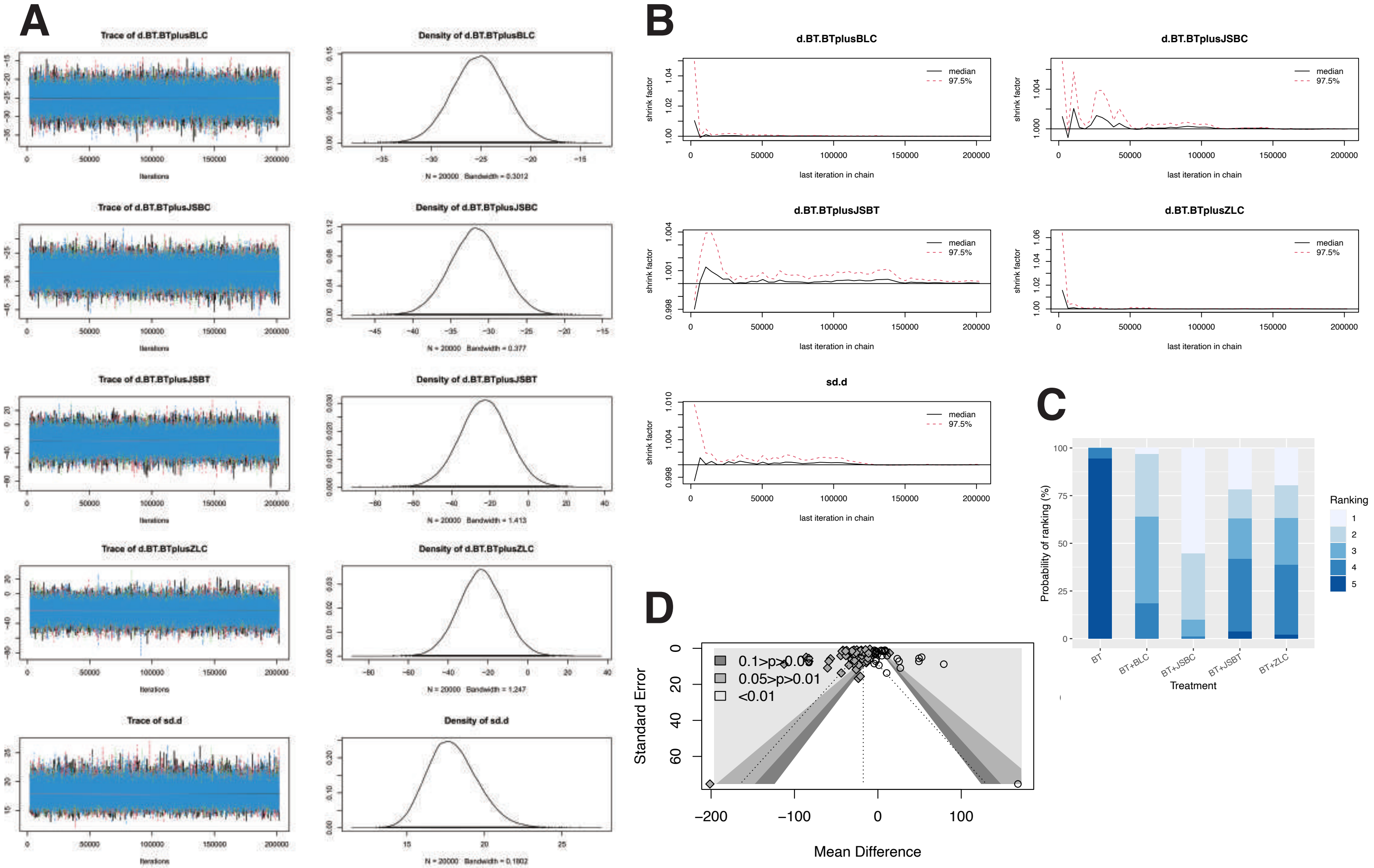

eFig.2b Urinary albumin excretion rate

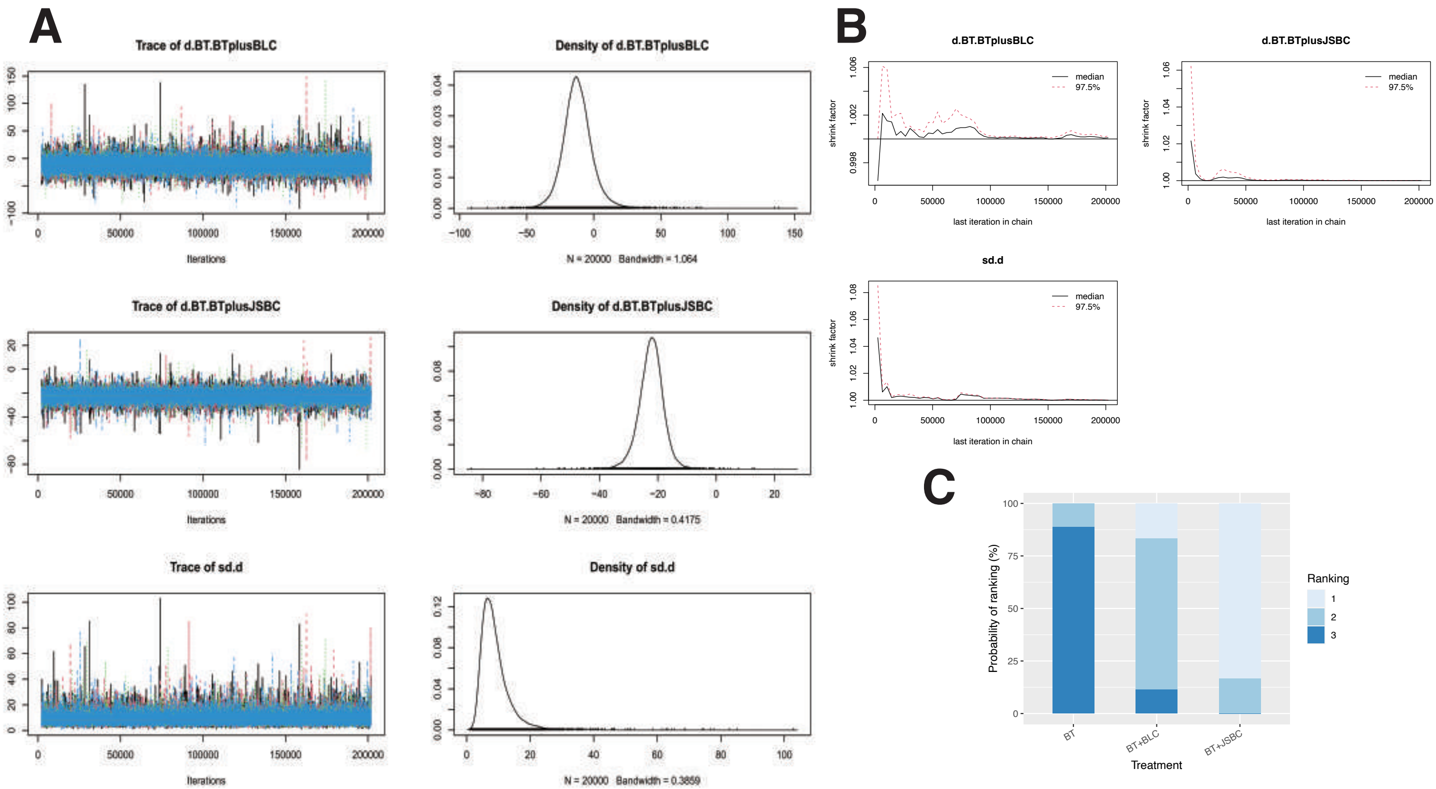

eFig.2c Urinary albumincreatinine ratio

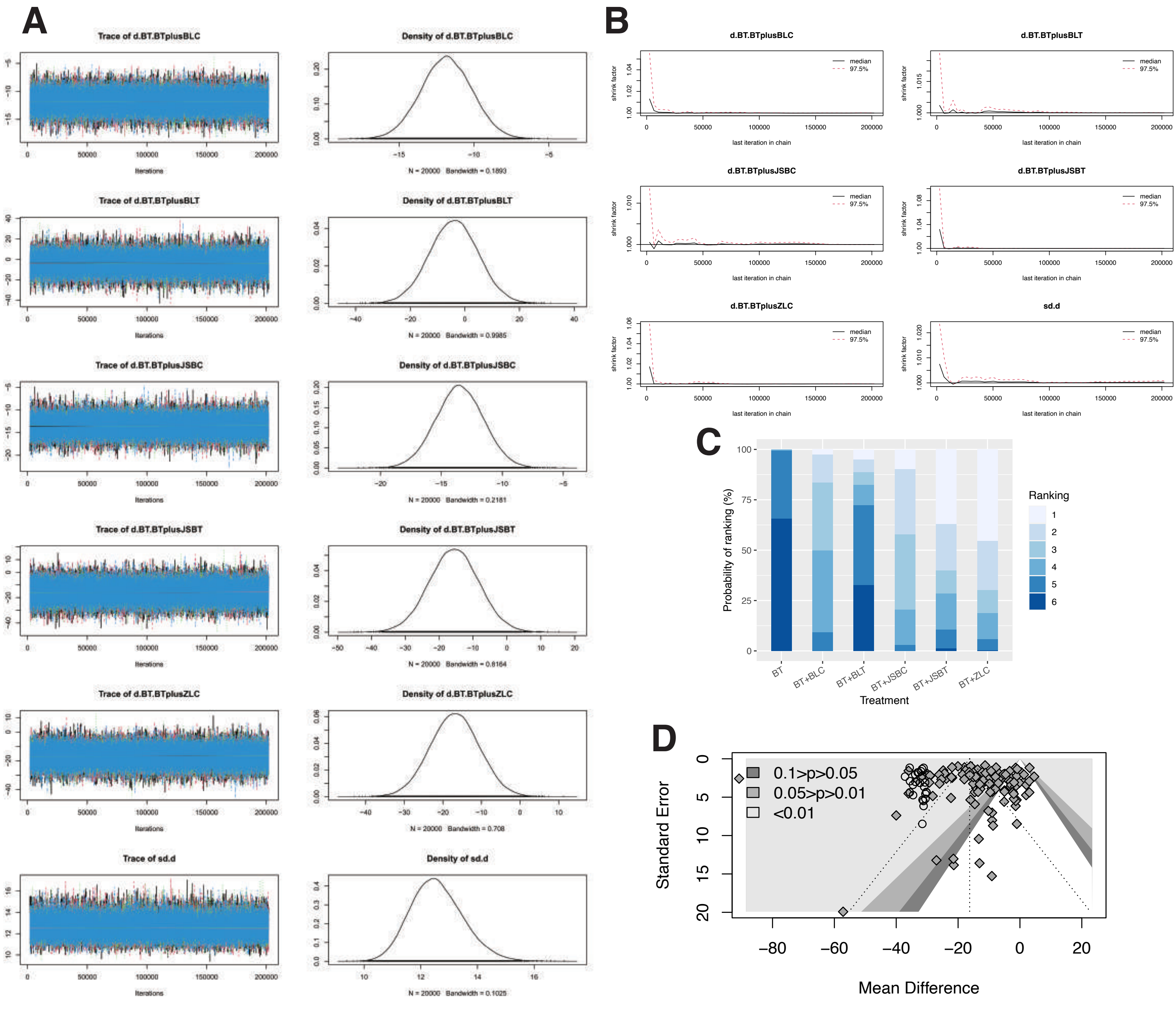

eFig.2d Serum creatinine

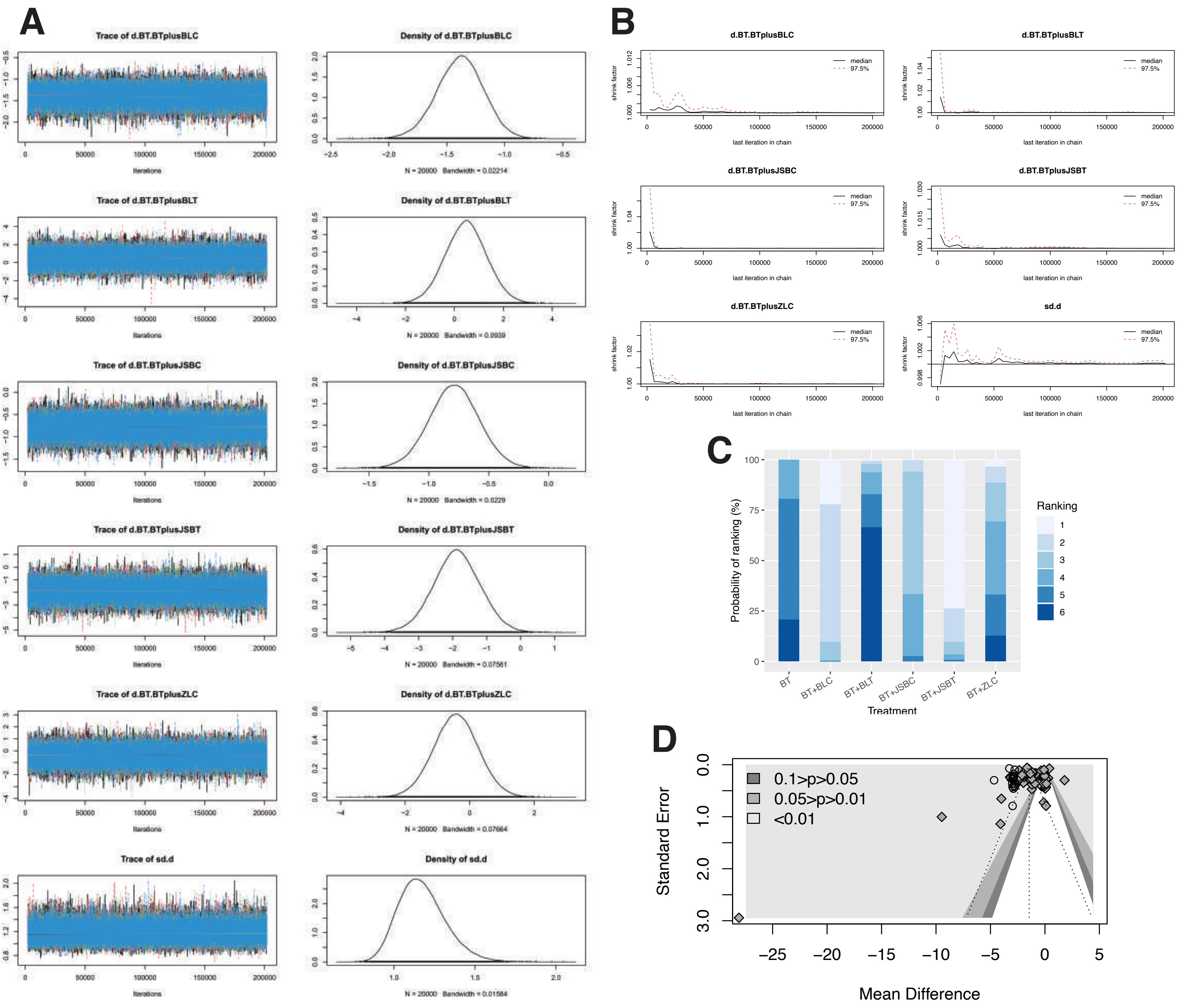

eFig.2e Serum urea nitrogen

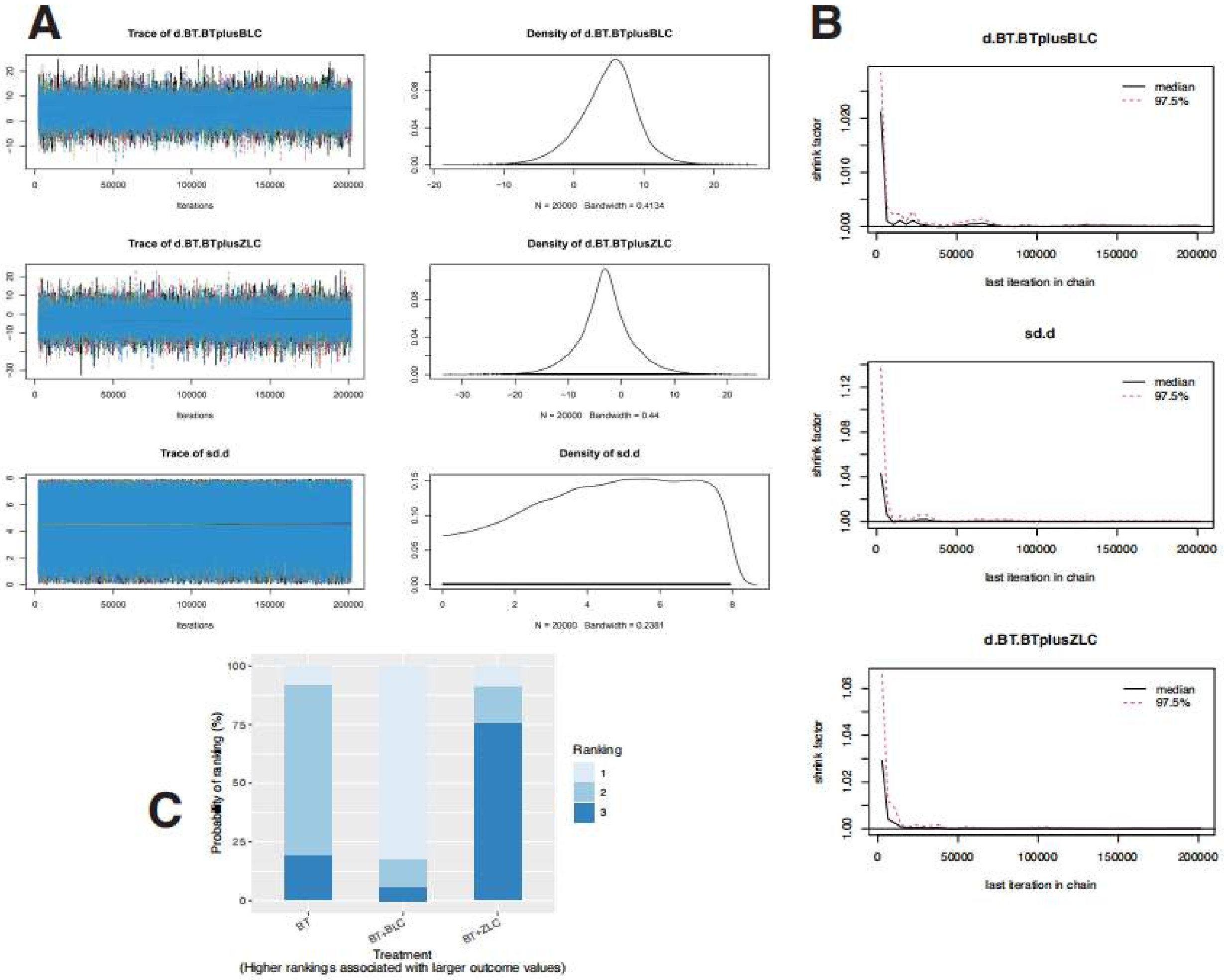

eFig.2f Estimated glomerular filtration rate

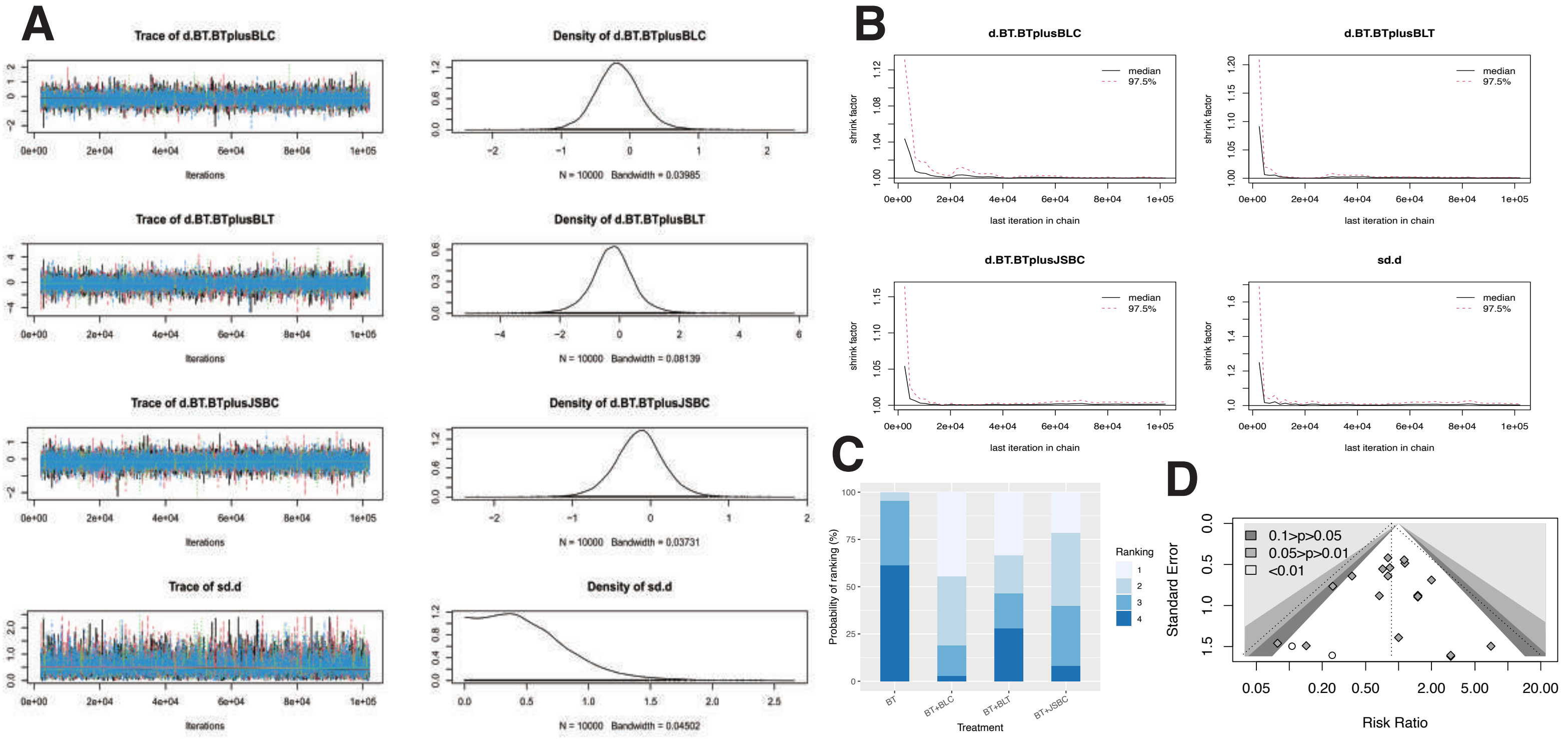

eFig.2g Adverse events

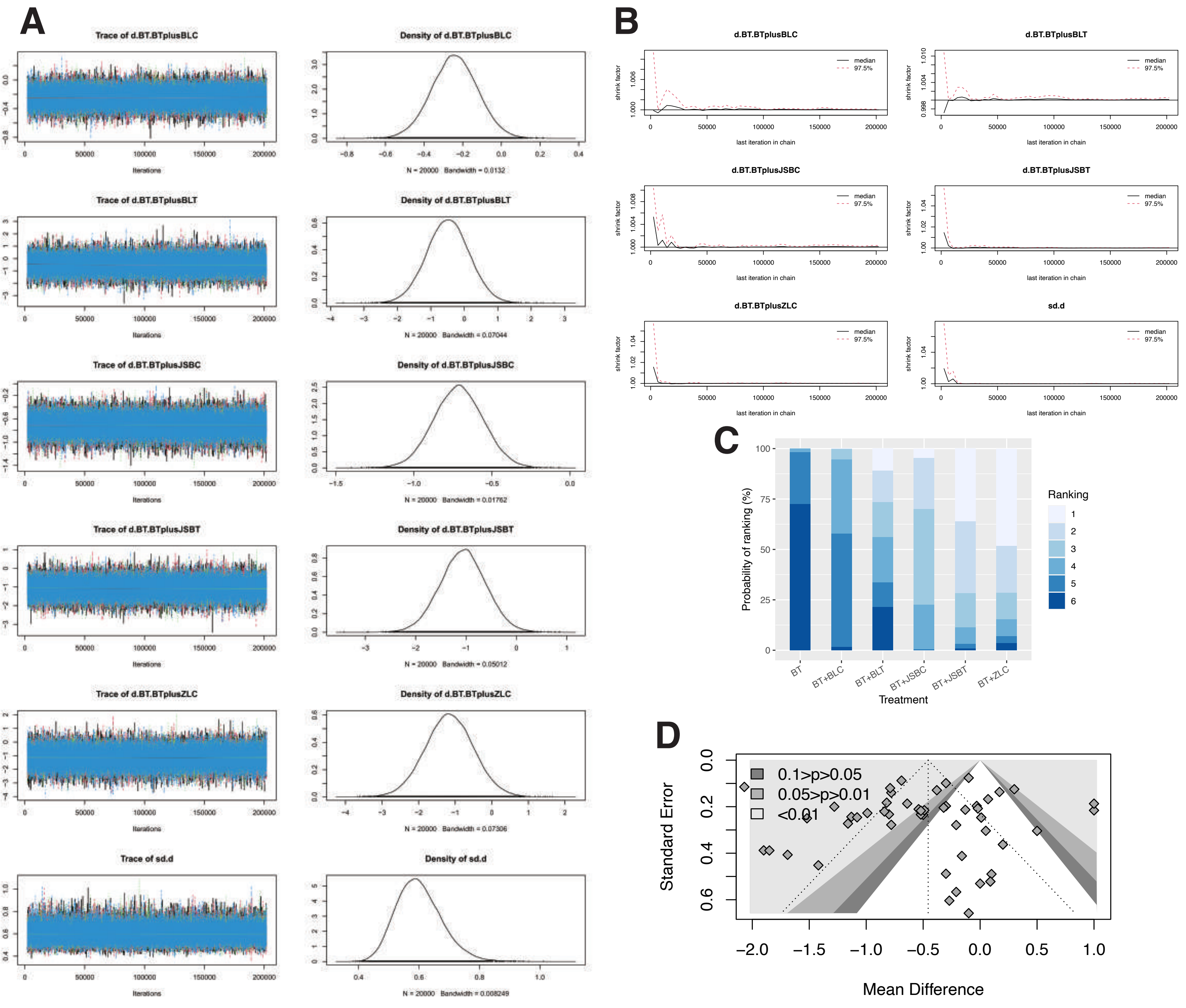

eFig.2h Fasting plasma glucose

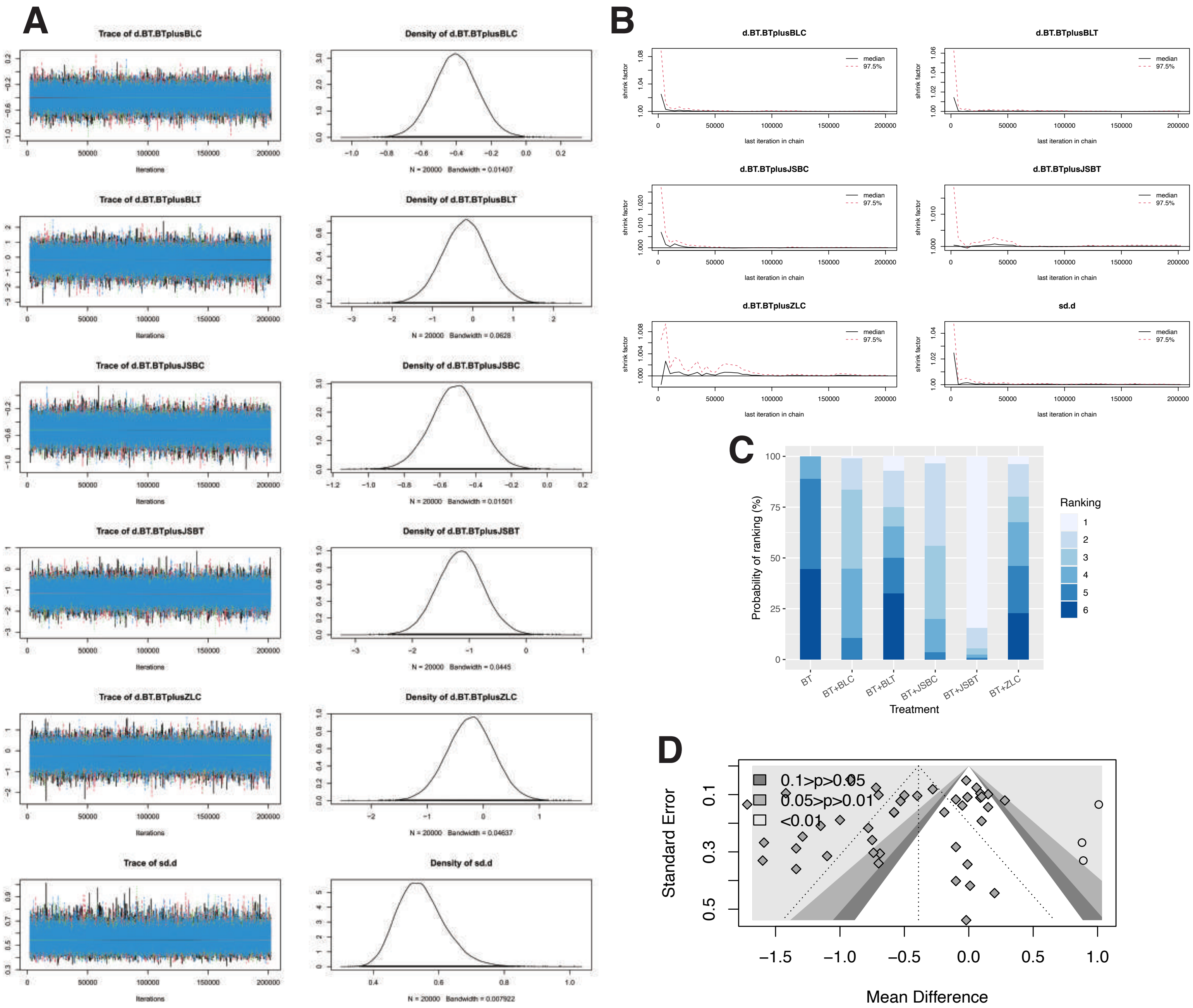

eFig.2i Glycated hemoglobin A1c
