# Supplementary material for: Ophiocordyceps sinensis preparations combined with the renin–angiotensin system inhibitor for diabetic kidney disease treatment: an umbrella review of systematic reviews and network meta-analysis
Source: Front Pharmacol. 2024 Apr 22;15:1360633. doi: 10.3389/fphar.2024.1360633 (PMC11075507; doi:10.3389/fphar.2024.1360633)
Supplement: Supplementary file 7 [file Table2.DOCX]

Supplementary Material

*Ophiocordyceps sinensis* preparations combined with renin-angiotensin system inhibitor for diabetic kidney disease: an umbrella review of systematic reviews and network meta-analysis

**Xue Xue^1^****^†^, Xin-yan Jin^2†^, Xing-lan Ye^3^, Ke-ying Li^3^, Jia-xuan Li^3^, Xue-han Liu^2^, Juan Bai^3^, Qiang Liu^4^, Bing-rui Zhang^5^, Xin-rong Zou^4^, Jun Yuan^6^, Chun-li Lu^7^, Fang-fang Zhao^8^, Jian-ping Liu^2^* and Xiao-qin Wang^4^***

*** Correspondence:**Jian-ping Liu: Liujp@bucm.edu.cn

Xiao-qin Wang: wangxiaoqin773@hotmail.com

# Supplementary Table 2A The list of excluded systematic reviews during full-text screening process

| **No.** | **Study ID** | **Title** | **The reason for exclusion** |
| --- | --- | --- | --- |
| 1 | He XH 2012 | Meta-analysis of the efficacy of Bailing capsule in the prevention and treatment of diabetic nephropathy | ACEIs/ARBs were not included in the intervention measures. |
| 2 | Huang YL 2012 | Systematic review on Bailing capsule for diabetic nephropathy early stage | ACEIs/ARBs were not included in the intervention measures. |
| 3 | Mao JX 2012 | Systematic review on Jinshuibao for diabetic nephropathy | ACEIs/ARBs were not included in the intervention measures. |
| 4 | Zhang YM 2012 | Jinshuibao capsule for diabetic nephropathy: A systematic review | ACEIs/ARBs were not included in the intervention measures. |
| 5 | Tang R 2013 | Systematic review on Bailing capsule combined with conventional treatment for diabetic nephropathy early stage | ACEIs/ARBs were not included in the intervention measures. |
| 6 | Wang QF 2013 | Clinical meta-analysis of artificial *cordyceps* preparations for intervention in type 2 diabetic nephropathy early stage | *Cordyceps* preparations combined with other Chinese medicine included in the intervention measures. |
| 7 | Ji XX 2014 | Systematic review on Bailing capsule for renal functional levels of diabetic nephropathy | ACEIs/ARBs were not included in the intervention measures. |
| 8 | Chen RC 2017 | Systematic review on *Cordyceps Sinensis* for diabetic nephropathy | *Cordyceps* preparations combined with other Chinese medicine included in the intervention measures. |
| 9 | Jing YF 2017 | Systematic review on *Cordyceps Sinensis* for diabetic nephropathyⅢ-Ⅳstages | *Cordyceps* preparations combined with other Chinese medicine included in the intervention measures. |
| 10 | Wen L 2018 | Patent of *Ophiocordyceps Sinensis* (Jinshuibao) for diabetic kidney disease: A systematic reviewand meta-analysis | ACEIs/ARBs were not included in the intervention measures. |
| 11 | Liu DD 2020 | *Cordyceps* preparation combined with angiotensin receptor blocker for diabetic nephropathy: A meta-analysis | *Cordyceps* preparations combined with other Chinese medicine included in the intervention measures. |
| 12 | Sheng XH 2020 | Efficacy and safety of Bailing capsules in the treatment of type 2 diabetic nephropathy: a meta-analysis | ACEIs/ARBs were not included in the intervention measures. |
| 13 | Gao Y 2021 | Adjunctive effect of Bailing capsule for diabetic kidney disease: A meta-analysis | ACEIs/ARBs were not included in the intervention measures. |
| 14 | Su F 2021 | Bailing capsule combined with liraglutide for diabetic nephropathy: A meta-analysis | ACEIs/ARBs were not included in the intervention measures. |
| 15 | Yu XY 2022 | Efficacy and Safety of Jinshuibao Capsule in Diabetic Nephropathy: A Systematic Review and Meta-Analysis of Randomized Controlled Trials | ACEIs/ARBs were not included in the intervention measures. |

**Abbreviation:** ACEIs/ARBs, angiotensin converting enzyme inhibitors/angiotensin receptor blockers.

# Supplementary Table 2B The list of excluded randomized controlled trials during full-text screening process

| **No.** | **Study ID** | **Title** | **The reason for exclusion** |
| --- | --- | --- | --- |
| 1 | Liu LZ 1999 | Clinical observation on the treatment of diabetic nephropathy with Bailing capsule | The random method was incorrect. |
| 2 | Wang L 2007 | Effect of combined treatment with Losartan and Bailing capsule on urinary microalbumin in early type 2 diabetic nephropathy in the elderly | The patent name was miswritten. No response was received after contacting the corresponding author. |
| 3 | Hong RT 2008 | Observation on the treatment of diabetic nephropathy with Irbesartan jointed by Zhiling capsule | The grouping was not random. |
| 4 | Guan X 2009 | Effects of Bailing capsule on cellular immunity in patients with diabetic nephropathy early stage | The primary and secondary outcome measures specified in our study protocol were not found. |
| 5 | Cui Y 2010 | Clinical study on angiotensin-converting enzyme inhibitor combined with Jinshuibao capsule for renal protection in patients with diabetic nephropathy | The dosages of ACEIs were different between the control and intervention groups. |
| 6 | Fang YY 2010 | Observation on the treatment of early diabetic nephropathy with Telmisartan jointed by Jinshuibao capsule | The random method was incorrect. |
| 7 | Yang SX 2010 | Effects of Bailing capsule on TNF-α levels for diabetic nephropathy | The primary and secondary outcome measures specified in our study protocol were not found. |
| 8 | Zhang LF 2010 | Jinshuibao capsule combined with Candesartan Cilexetil tablets for the treatment of early type 2 diabetic nephropathy in 40 cases | The grouping was not random. |
| 9 | Ge QR 2011 | The efficiency of Benazepril Hydrochloride combined with Jinshuibao capsule for diabetic nephropathy | The random method was incorrect. |
| 10 | Liu YH 2011 | Effect of Irbesartan combined with Bailing capsule on microalbuminuria in patients with early diabetic nephropathy | The grouping was not random. |
| 11 | Li ZH 2012 | Observations on the efficacy of combined medication for diabetic nephropathy | All outcomes have no unit of measure.No response was received after contacting the corresponding author. |
| 12 | Luo F 2011 | Clinical studies of adjuvant therapeutic effects of Bailing capsule for early diabetic nephropathy | ACEIs/ARBs were not included in basic treatment. |
| 13 | Guo JZ 2012 | Effect observation on the treatment of type 2 diabetes with early stage nephropathy by Irbesartan combined with Jinshuibao capsule | The grouping was not random. |
| 14 | Wu L 2012 | Clinical observation on 100 cases of early diabetic nephropathy patients treated with Bailing capsule | The primary and secondary outcome measures specified in our study protocol were not found. |
| 15 | Yang L 2012 | Effect of Bailing capsule on oxidative stress levels with early diabetic nephropathy | The random method was incorrect. |
| 16 | Yang XM 2012 | Clinical observation on the treatment of diabetic nephropathy with Enalapril combined with Jinshuibao capsule | All outcomes have no unit of measure. No response was received after contacting the corresponding author. |
| 17 | Bu SS 2013 | Valsartan combined with Jinshuibao capsule for microinflammatory state in early diabetic nephropathy | The random method was incorrect. |
| 18 | Li HJ 2013 | Clinical application of Bailing capsule combined with Irbesartan in early diabetic nephropathy | The primary and secondary outcome measures specified in our study protocol were not found. |
| 19 | Li YN 2013 | Irbesartan combined with Jinshuibao capsule for early diabetic nephropathy | The number of men plus women is more than the total number. No response was received after contacting the corresponding author. |
| 20 | Chen L 2014 | Clinical observation on the treatment of early diabetic nephropathy with Valsartan jointed by Bailing capsule | The primary and secondary outcome measures specified in our study protocol were not found. |
| 21 | Lu DH 2014 | Clinical efficacy observation on Valsartan combined with Jinshuibao for diabetic nephropathy | The random method was incorrect. |
| 22 | Wang TP 2014 | Randomized parallel controlled study Jinshuibao and Enalapril in the treatment of diabetic nephropathy | The primary and secondary outcome measures specified in our study protocol were not found. |
| 23 | Xiang JS 2014 | Clinical analysis on 60 cases of early diabetic nephropathy treated by Jinshuibao capsule combined with Olmesartan Medoxomil | The random method was incorrect. |
| 24 | Fan YT 2015 | Clinical observation on Candesartan and Jinshuibao capsule for early diabetic nephropathy | The primary and secondary outcome measures specified in our study protocol were not found. |
| 25 | Shen ML 2015 | Observation on Candesartan combined with Jinshuibao capsule for early type 2 diabetic nephropathy | The random method was incorrect. |
| 26 | Sun JY 2015 | Study on Jinshuibao capsule combined with Losartan Potassium tablets for diabetic nephropathy early stage | The dosages of ACEIs were different between the control and intervention groups. |
| 27 | Wang XH 2015 | Clinical observation on 60 cases of early diabetic nephropathy treated by Valsartan combined with Bailing capsule | The grouping was not random. |
| 28 | Yan ZH 2015 | Discussion and analysis of combining Valsartan and Jinshuibao capsule for diabetic nephropathy | The random method was incorrect. |
| 29 | Chen ZG 2016 | Effect of Bailing capsule combined with Irbesartan on blood glucose, blood pressure and blood lipids of patients with diabetic nephropathy | The random method was incorrect. |
| 30 | Guo T 2016 | Observation on the curative effect of Benazepril Hydrochloride tablet combined with Jinshuibao capsule for early diabetic nephropathy | The random method was incorrect. |
| 31 | Cai WJ 2017 | Effect observation on early type 2 diabetic nephropathy by Jinshuibao combined with Olmesartan Medoxomil | The random method was incorrect. |
| 32 | Yan TR 2017 | Clinical analysis of early diabetic nephropathy with Valsartan combined with Jinshuibao capsule | The random method was incorrect. |
| 33 | Zhang YS 2017 | Clinical effect observation on treatment of type 2 diabetes patients with early stage nephropathy by Irbesartan and Jinshuibao | The primary and secondary outcome measures specified in our study protocol were not found. |
| 34 | Zhu J 2017 | Study on the effects of Jinshuibao capsule combined with Valsartan on inflammatory factors, blood pressure and renal function in patients with type 2 diabetic nephropathy | The random method was incorrect. |
| 35 | Li Y 2018 | Clinical observation on Jinshuibao capsule combined with Western Medicine for early diabetic nephropathy | The grouping was not random. |
| 36 | Wang LW 2018 | Effect analysis of Candesartan Cilexetil tablets combined with Jinshuibao for type 2 diabetic nephropathy early stage | The random method was incorrect. |
| 37 | Zhang D 2018 | Observation on Bailing capsule combined with Losartan in the treatment of early diabetic nephropathy and its effect on patients' urinary micro-protein and CRP levels | The grouping was not random. |
| 38 | Cao YX 2019 | Clinical observation on the treatment of early diabetic nephropathy with Benazepril combined with Jinshuibao capsule | The random method was incorrect. |
| 39 | Ding Y 2019 | The effect of Enalapril combined with Bailing capsules on renal function and insulin resistance index levels in patients with type 2 diabetic nephropathy | The grouping was not random. |
| 40 | Fang C 2019 | Effect of Bailing capsule combined with Enalapril on blood glucose and renal function in elderly diabetic nephropathy patients | The random method was incorrect. |
| 41 | Li Q 2019 | Clinical application of Bailing capsule combined with Losartan for early diabetic nephropathy | The random method was incorrect. |
| 42 | Li Y 2019 | Efficacy and safety of Bailing capsule combined with Candesartan for diabetic nephropathy | The random method was incorrect. |
| 43 | Ren LJ 2019 | Clinical observation on early diabetic nephropathy with Jinshuibao capsule combined with Western Medicine | The random method was incorrect. |
| 44 | Wei JW 2019 | Effects of Bailing capsule combined with high-dose Ramipril on renal function and glycemic control in patients with early diabetic nephropathy | The grouping was not random. |
| 45 | Jiang JC 2020 | Clinical efficacy of Bailing capsule combined with Simvastatin in the treatment of type 2 diabetic nephropathy | The random method was incorrect. |
| 46 | Li Q 2020 | Clinical effect analysis of early diabetic nephropathy treated with Bailing capsule | ACEIs/ARBs were not included in the intervention group. |
| 47 | Liu GY 2020 | Preliminary efficacy and safety analysis of Jinshuibao capsule on men with diabetic nephropathy with sexual dysfunction | The primary and secondary outcome measures specified in our study protocol were not found. |
| 48 | Sun GW 2020 | Efficacy of Valsartan combined with Jinshuibao capsule for diabetic nephropathy with sexual dysfunction | The primary and secondary outcome measures specified in our study protocol were not found. |
| 49 | Wang AX 2020 | Clinical study of valsartan combined with Jinshuibao capsule for sexual dysfunction complicated by diabetic nephropathy | The primary and secondary outcome measures specified in our study protocol were not found. |
| 50 | Wen QMTHT 2020 | Exploring the efficacy and safety of the traditional Chinese medicine *Cordyceps Sinensis* for diabetic nephropathy | Bailing capsule and Jinshuibao capsule were included the intervention group. |
| 51 | Yang YP 2020 | Clinical value of 72 patients with early diabetic nephropathy treated with Bailing capsule combined with Valsartan | The random method was incorrect. |
| 52 | Zhang Q 2020 | Clinical observation on early diabetic nephropathy with Bailing capsule combined with Valsartan | The random method was incorrect. |
| 53 | Gao J 2021 | Clinical observation on Jinshuibao tablet for early diabetic nephropathy | ACEIs/ARBs were not included in basic treatment. |
| 54 | Ning JY 2021 | Clinical study of diabetic nephropathy treated with Bailing capsule combined with valsartan | The primary and secondary outcome measures specified in our study protocol were not found. |
| 55 | Wang XJ 2021 | Observation on the treatment of early diabetic nephropathy with Jinshuibao capsule | The random method was incorrect. |
| 56 | Wang Y 2021 | Clinical study on urinary micro-protein and inflammatory mediator levels in patients with diabetic nephropathy by combination of Bailing capsule and Losartan | The random method was incorrect. |
| 57 | Xin ZP 2021 | The effect of Bailing capsule on urinary microalbumin in the treatment of diabetic nephropathy | ACEIs/ARBs were not included in the intervention group. |
| 58 | Yang YJ 2021 | Clinical effect of Enalapril combined with Jinshuibao capsule in the treatment of diabetic nephropathy | ACEIs/ARBs were not included in the control group. |
| 59 | Yang Y 2021 | Observation on the effect of Bailing capsule combined with Telmisartan for patients with diabetic nephropathy | The grouping was not random. |
| 60 | Zuo JJ 2022 | Clinical efficacy of Tripterygium glycosides tablet combined with Bailing capsule for patients with stage Ⅳ diabetic nephropathy | The grouping was not random. |
| 61 | Li HN 2022 | Effect of RAAS blocker combined with Bailing capsule on early diabetic nephropathy and blood glucose levels | The grouping was not random. |
| 62 | Wang Y 2022 | Analysis of Bailing capsule combined with Losartan for the treatment of early diabetic nephropathy | The random method was incorrect. |
| 63 | Wu MJ 2022 | Effectiveness of Bailing capsule combined with valsartan capsule for type 2 diabetic nephropathy and the mechanism of maintaining oxidative homeostasis | The grouping was not random. |
| 64 | Xu LN 2023 | Clinical observation on Jinshuibao capsule combined with Irbesartan for diabetic nephropathy | The unit of measurement of ACR was incorrect. |

**Abbreviation:** ACEIs/ARBs, angiotensin converting enzyme inhibitors/angiotensin receptor blockers; ACR, albumin/creatinine ratio.
