# Supplementary material for: Ophiocordyceps sinensis preparations combined with the renin–angiotensin system inhibitor for diabetic kidney disease treatment: an umbrella review of systematic reviews and network meta-analysis
Source: Front Pharmacol. 2024 Apr 22;15:1360633. doi: 10.3389/fphar.2024.1360633 (PMC11075507; doi:10.3389/fphar.2024.1360633)
Supplement: Supplementary file 8 [file Table5.DOCX]

Supplementary Material

*Ophiocordyceps sinensis* preparations combined with renin-angiotensin system inhibitor for diabetic kidney disease: an umbrella review of systematic reviews and network meta-analysis

**Xue Xue^1^****^†^, Xin-yan Jin^2†^, Xing-lan Ye^3^, Ke-ying Li^3^, Jia-xuan Li^3^, Xue-han Liu^2^, Juan Bai^3^, Qiang Liu^4^, Bing-rui Zhang^5^, Xin-rong Zou^4^, Jun Yuan^6^, Chun-li Lu^7^, Fang-fang Zhao^8^, Jian-ping Liu^2^* and Xiao-qin Wang^4^***

*** Correspondence:**Jian-ping Liu: Liujp@bucm.edu.cn

Xiao-qin Wang: wangxiaoqin773@hotmail.com

# Supplementary Table 5 Characteristics of each included randomized controlled trial

| Study ID | Sample-size (E/C) | Age/(year) (E/C) | | Sex (Male/Female) (E/C) | | Stage of DKD patients | Intervention (E/C) | | Course of treatment | Dosage and frequency of intervention (E/C) | | Outcomes |
| --- | --- | --- | --- | --- | --- | --- | --- | --- | --- | --- | --- | --- |
| Jia ZR 1999 | 18/20 | 55±2.2 | 54±4.4 | 10/8 | 12/8 | Early | BLC + BT | BT (Captopril) | 6 w | BLC 5 capsules tid | Captopril 12.5mg bid/tid | (1) |
| Yan Y 2005 | 40/20 | 49.7±8.9 | 50.2±9.1 | 22/18 | 12/8 | Ⅲ | BLC + BT | BT (Enalapril) | 8 w | BLC 5 capsules tid | Enalapril 10mg qd | (1)(10) |
| Lv H 2006 | 48/32 | 49.5±9.5 | 48.6±8.9 | 27/21 | 17/15 | NR | JSBC + BT | BT (Valsartan) | 16 w | NR | | (1) |
| Cao XX 2007 | 30/30 | 59.43±14.14 | 60.21±10.32 | 15/15 | 13/17 | NR | JSBC + BT | BT (Valsartan) | 6 m | JSBC 6 capsules tid | Valsartan 80mg qd | (1)(5)(8) |
| Chen Y 2007 | 30/30 | 58.50±10.89 | 59.3±7.29 | 18/12 | 16/14 | Ⅲ | BLC + BT | BT (Fosinopril) | 8 w | BLC 3 capsules tid  (0.29g/capsule) | Fosinopril 10mg qd | (1)(6)(7)(8)(10) |
| Wang HP 2007 | 35/33 | 58.3±3.6 | 57.6±3.8 | 18/17 | 17/16 | Early | JSBC + BT | BT (Fosinopril) | 6 w | JSBC 3 capsules tid | Fosinopril 10mg qd | (1) |
| Wang YH 2008 | 30/30 | NR | | NR | | Ⅲ | BLC + BT | BT (Telmisartan) | 3 m | BLC 5 capsules tid | Telmisartan 40mg qd | (1)(7)(8) |
| Wu ZF 2008 | 25/25 | Total 46~74 | | Total 28/22 | | Early | JSBC + BT | BT (Irbesartan) | 6 w | JSBC 6 capsules tid | Irbesartan 150mg qd | (5) |
| Chen J 2009 | 43/43 | 59.3±7.8 | 58.5±8.0 | 28/15 | 27/16 | Early | BLC + BT | BT (Valsartan) | 12 w | BLC 5 capsules tid  (0.2g/capsule) | Valsartan 160mg qd | (1)(2)(4)(6)(7) |
| Cui TX 2009 | 25/25 | 53±11 | 51±12 | 13/12 | 15/10 | NR | BLC + BT | BT (Telmisartan) | 6 m | BLC tid  (1.0g/capsule) | Telmisartan 80mg qd | (3)(6) |
| Lei SH 2009 | 47/45 | Total 59.8±7.1 | | Total 56/36 | | Early | JSBC + BT | BT (Irbesartan) | 24 w | JSBC tid  (1.98g/capsule) | Irbesartan 150mg qd | (1)(2)(7)(8) |
| Li JE 2009 | 30/30 | 67.0±4.8 | 66.0±3.5 | 17/13 | 14/16 | Ⅲ-Ⅳ | BLC + BT | BT (Valsartan) | 8 w | BLC 5 capsules tid | Valsartan 80mg qd | (2)(3)(4) |
| Lin J 2009 | 18/18 | 48.9±17.5 | 50.7±16.4 | 9/9 | 10/8 | Ⅳ | JSBC + BT | BT (Valsartan) | 8 w | JSBC 3 capsules tid | Valsartan 80mg qd | (2)(3)(4) |
| Song J 2009 | 30/30 | 51.2±17.2 | 50.3±16.7 | 16/14 | 18/12 | Ⅲ | BLC + BT | BT (Benazepril) | 16 w | BLC 2.0g tid | Benazepril 10mg qd | (1)(2)(3)(7)(8) |
| Tao SC 2009 | 43/42 | 50.6±6.7 | 51.2±6.9 | 23/20 | 21/21 | Early | JSBC + BT | BT (Enalapril) | 6 m | JSBC 3 capsules tid | Enalapril 10mg qd | (2)(6) |
| Wang SY 2009 | 25/25 | NR | | NR | | Ⅲ | BLC + BT | BT (Benazepril) | 3 m | BLC 5 capsules tid | Benazepril 10mg qd | (1)(2)(7)(8) |
| Xue XH 2009 | 31/33 | 63±5 | 62±6 | 16/15 | 17/16 | NR | BLC + BT | BT (Irbesartan) | 12 w | BLC 5 capsules tid | Irbesartan 150mg qd | (1)(2)(7) |
| Yi JZ 2009 | 20/20 | 67.0±4.8 | 66.2±5.4 | 8/12 | 13/7 | Ⅲ-Ⅳ | JSBC + BT | BT (Irbesartan) | 12 w | JSBC 3 capsules tid | Irbesartan150mg qd | (2)(3)(4) |
| Zhang ZF 2009 | 44/41 | NR | | NR | | Early | BLC + BT | BT (Benazepril Hydrochloride) | 3 m | BLC 5 capsules tid | Benazepril Hydrochloride 10mg qd | (1)(2)(4)(8)(10) |
| Chen F 2010 | 28/26 | Total 36~73 | | Total 26/28 | | Ⅲ | BLC + BT | BT (Valsartan) | 8 w | BLC 1.0g tid | Valsartan 80mg bid | (1)(7) |
| Guan HB 2010 | 31/31 | 52.2±6.5 | 53.3±5.7 | Total 33/29 | | Ⅲ | BLC + BT | BT (Irbesartan) | 24 w | BLC 5 capsules tid  (0.2g/capsule) | Irbesartan 150mg qd | (2)(7)(8) |
| Hong YQ 2010 | 30/30 | Total 52~72 | | Total 21/39 | | Ⅲ-Ⅳ | BLC + BT | BT (Irbesartan) | 16 w | BLC 5 capsules tid | Irbesartan 150mg qd | (2)(3)(4) |
| Huang T 2010 | 32/32 | Total 60~85 | | Total 49/15 | | Ⅲ | JSBC + BT | BT (Perindopril) | 12 w | JSBC tid  (1.98g/capsule) | Perindopril 4mg qd | (1)(3)(7)(8) |
| Liu Y 2010 | 40/40 | 41.2±5.4 | 43.8±5.4 | Total 44/36 | | Ⅲ-Ⅳ | JSBC + BT | BT (Telmisartan) | 16 w | JSBC tid  (0.2g/capsule) | Telmisartan 80mg qd | (2)(3)(4) |
| Li XL 2010 | 42/36 | Total 55.7±9.6 | | Total 38/40 | | Ⅲ | BLC + BT | BT (Losartan) | 4 w | BLC 5 capsules tid | Losartan 100mg qd | (1)(4)(6)(7) |
| Lou PH 2010 | 31/31 | 61±10 | 60±8 | 16/15 | 15/16 | Early | BLC + BT | BT (Valsartan) | 4 w | BLC 1.0g tid | Valsartan 80mg qd | (2)(4)(6) |
| Lu YL 2010 | 33/33 | NR | | NR | | NR | BLC + BT | BT (Telmisartan) | 4 w | BLC 1.0g tid | Telmisartan 80mg qd | (2) |
| Shi HB 2010 | 37/35 | 58.1±3.5 | 57.8±3.7 | Total 36/36 | | Early | JSBC + BT | BT (Perindopril) | 8 w | JSBC 3 capsules tid | Perindopril 4mg qd | (1) |
| Wang YZ 2010 | 30/30 | 64.9±4.6 | 66.0±3.5 | 17/13 | 14/16 | Early | BLC + BT | BT (Benazepril Hydrochloride) | 12 w | BLC 5 capsules tid | Benazepril Hydrochloride 10mg qd | (2)(3)(4) |
| Wei SJ 2010 | 30/30 | Total 37~70 | | Total 38/22 | | Ⅲ | JSBC + BT | BT (Benazepril) | 8 w | JSBC 5 capsules tid | Benazepril 10mg qd | (2)(3)(4) |
| Xiong D 2010 | 30/30 | Total 51.7±2.1 | | Total 66/54 | | Ⅳ | JSBC + BT | BT (Irbesartan) | 12 m | JSBC 3 capsules tid | Irbesartan150mg qd | (1)(2)(4) |
| Zeng SX 2010 | 39/39 | 68.4±5.1 | 67.5±4.7 | Total 43/35 | | Early | JSBC + BT | BT (Irbesartan) | 6 w | JSBC 6 capsules tid | Irbesartan 150mg qd | (3)(5)(7) |
| Chen WJ 2011 | 50/50 | Total 48.0±14.8 | | Total 48/52 | | Early | ZLC + BT | BT (Irbesartan) | 6 m | ZLC 3 capsules tid | Irbesartan 150mg qd | (1)(2)(4)(7)(8) |
| Li H 2011 | 23/23 | 38~66 | 42~69 | 14/9 | 10/13 | Ⅳ | BLC + BT | BT (Irbesartan) | 3 m | BLC 5 capsules tid | Irbesartan 300mg | (2)(3)(6) |
| Liu CP 2011(1) | 32/32 | 54±17 | 52±14 | 15/17 | 13/19 | Ⅲ | BLC + BT | BT (Irbesartan) | 12 w | BLC 1.0g tid | Irbesartan 150mg qd | (2)(3)(4)(7) |
| Liu CP 2011(2) | 32/32 | Total 54±17 | | 15/17 | 13/19 | Ⅲ | BLC + BT | BT (Irbesartan) | 12 w | BLC 1.0g tid | Irbesartan 150mg qd | (1)(2)(6)(7) |
| Liu MW 2011 | 20/20 | Total 35~72 | | Total 21/19 | | Early | BLC + BT | BT (Losartan) | 16 w | BLC 9.0g qd | Losartan 50mg qd | (1)(2) |
| Liu XD 2011 | 79/79 | 57.7±5.3 | NR | 48/31 | NR | NR | BLC + BT | BT (Benazepril) | 2 m | BLC 6 capsules tid | Benazepril 10mg qd | (1)(2)(3)(4) |
| Luo F 2011 | 40/40 | NR | | NR | | Ⅲ | BLC + BT | BT (Irbesartan) | 90 d | BLC 5 capsules tid | Irbesartan 150mg qd | (1)(2) |
| Ma YL 2011 | 25/25 | 46.2±7.9 | 48.1±9.3 | 13/12 | 12/13 | Early | BLC + BT | BT (Irbesartan) | 12 w | BLC 1.0g tid | Irbesartan 150mg qd | (1)(2)(8) |
| Shen YL 2011 | 40/40 | NR | | NR | | NR | BLC + BT | BT (Valsartan) | 12 w | BLC 5 capsules tid | Valsartan 80~160mg qd | (3) |
| Tang YZ 2011 | 40/40 | 59.2±3.8 | 57.7±4.2 | 23/17 | 24/16 | Early | JSBC + BT | BT (Telmisartan) | 12 w | JSBC 3 capsules tid | Telmisartan 20~40mg qd | (1)(2)(7)(8) |
| Wang RF 2011 | 40/40 | 32~65 | 34~66 | 25/15 | 28/13 | NR | JSBC + BT | BT (Benazepril Hydrochloride) | 12 w | JSBC 6 capsules tid | Benazepril Hydrochloride 10mg qd | (2)(3)(4)(6) |
| Wang Y 2011 | 48/48 | 61.8±9.5 | 64.7±8.7 | 20/28 | 26/22 | Ⅳ | BLC + BT | BT (Valsartan) | 90 d | BLC 10 capsules tid  (0.2g/capsule) | Valsartan 80mg qd | (2)(3)(4)(7)(8) |
| Xiong J 2011 | 30/30 | 36~60 | 35~60 | 15/15 | 14/16 | Ⅲ | BLC + BT | BT (Irbesartan) | 12 w | BLC 5 capsules tid | Irbesartan 150mg qd | (1) |
| Yang JQ 2011 | 30/30 | 66.1±4.5 | 65±3.5 | 15/15 | 16/14 | NR | JSBC + BT | BT (Benazepril Hydrochloride) | 12 w | JSBC 5 capsules tid | Benazepril Hydrochloride 10mg qd | (2)(3)(4) |
| Zhang YK 2011 | 42/42 | Total 68.4±5.1 | | Total 59/25 | | NR | JSBC + BT | BT (Irbesartan) | 6 w | JSBC 6 capsules tid | Irbesartan 150mg qd | (2)(3)(7) |
| Zheng JX 2011 | 32/32 | 47~63 | 45~71 | 15/17 | 19/13 | NR | BLC + BT | BT (Irbesartan) | 12 w | BLC 1.0g tid | Irbesartan 150mg qd | (1)(2)(4) |
| Cao LM 2012 | 18/18 | 51~79 | 51~77 | 7/11 | 9/9 | NR | BLC + BT | BT (Valsartan) | 12 w | BLC 1.0g tid | Valsartan 80mg qd | (1)(2)(4)(6) |
| He P 2012 | 43/43 | 56.6±8.4 | 57.4±8.9 | 28/15 | 24/19 | NR | JSBC + BT | BT (Valsartan) | 4 w | JSBC 3 capsules tid | Valsartan 80mg qd | (1)(2)(3)(4)(6)(7)(8) |
| Hu WF 2012 | 40/40 | 38~72 | 33~70 | 22/18 | 21/18 | Early | BLC + BT | BT (Irbesartan) | 16 w | BLC 2.0g tid  (0.2g/capsule) | Irbesartan 150mg qd | (1)(3)(7)(8) |
| Li QY 2012 | 50/50 | NR | | NR | | NR | BLC + BT | BT (Irbesartan) | 6 m | BLC 5 capsules tid | Irbesartan 150mg qd | (3) |
| Li X 2012 | 27/27 | Total 57.5±8.3 | | Total 31/23 | | NR | JSBC + BT | BT (Benazepril) | NR | JSBC 1.65g tid | Benazepril 20mg qd | (3) |
| Lv F 2012 | 28/28 | Total 36~57 | | Total 32/24 | | Ⅲ | JSBC + BT | BT (Enalapril) | 12 w | JSBC 3 capsules tid | Enalapril 10mg bid Irbesartan 150mg qd | (1)(2)(5)(6) |
| Lv ZM 2012 | 31/30 | 63.8±3.6 | 64.6±4.1 | 18/13 | 17/13 | Early | ZLC + BT | BT (Losartan Potassium) | 8 w | ZLC 2 capsules tid | Losartan Potassium 50mg qd | (1)(2)(4) |
| Qian GF 2012 | 40/40 | Total 61.74±7.21 | | Total 47/33 | | NR | JSBC + BT | BT (Enalapril) | 12 w | JSBC 3 capsules tid | Enalapril 10mg qd | (2)(3)(4) |
| Shan GH 2012 | 30/30 | 52±15 | 52±14 | 16/14 | 15/15 | Ⅳ | BLC + BT | BT (Valsartan) | 14 w | BLC 5 capsules tid | Valsartan 80mg qd | (2)(3)(4) |
| Shen SM 2012 | 46/40 | Total 65.2±15.8 | | Total 50/36 | | Ⅲ | BLC + BT | BT (Valsartan) | 12 w | BLC 1.0g tid | Valsartan 80~160mg qd | (1)(3) |
| Sun QH 2012 | 45/45 | 75.6±4.6 | 72.0±1.5 | 20/25 | 22/23 | Early | BLC + BT | BT (Enalapril Maleate) | 12 w | BLC 5 capsules tid | Enalapril Maleate  5mg bid | (2)(3)(4) |
| Wang W 2012 | 33/27 | Total 34~78 | | Total 40/20 | | NR | BLC + BT | BT (Captopril) | 6 m | BLC 1.0g tid | Captopril 25mg bid | (2)(3)(4)(7)(8) |
| Wu WT 2012 | 40/40 | 61.2±13.5 | 59.6±15.8 | 21/19 | 22/18 | Ⅲ | JSBC + BT | BT (Valsartan) | 12 m | JSB 6 capsules tid | Valsartan 80mg qd | (1)(2)(4) |
| Ye JB 2012 | 26/26 | 54±5 | 55±5 | 12/14 | 15/11 | Ⅲ | BLC + BT | BT (Candesartan Cilexetil) | 4 w | BLC 1.0g tid | Candesartan Cilexetil 8mg qd | (1)(2)(3) |
| Yu J 2012 | 42/42 | 64.3±4.2 | 63.8±4.4 | 27/15 | 26/16 | Early | BLC + BT | BT (Losartan Potassium) | 8 w | BLC 5 capsules tid | Losartan Potassium 50mg qd | (1)(2)(4) |
| Zhang Y 2012 | 30/30 | NR | | 15/15 | 16/14 | Early | JSBC + BT | BT (Valsartan) | 3 m | JSBC 5 capsules tid  (0.33g/capsule) | Valsartan 80mg qd | (1)(5)(8) |
| Zhou P 2012 | 29/29 | NR | | NR | | Early | JSBC + BT | BT (Losartan Potassium) | 4 w | JSBC 3 capsules tid | Losartan Potassium  50mg qd | (2) |
| Gao TW 2013 | 100/105 | 57.3±5.7 | 59.3±5.2 | 57/43 | 61/44 | Ⅲ | JSBC + BT | BT (Valsartan) | 12 w | JSBC 3 capsules tid  (0.33g/capsule) | Valsartan 80mg qd | (2)(3)(4) |
| Qiao AM 2013 | 62/62 | 55.9±11.8 | 54.6±12.4 | 32/30 | 33/29 | Ⅲ | BLC + BT | BT (Telmisartan) | 12 w | BLC 1.0g tid | Telmisartan 80mg qd | (1)(6) |
| Xie P 2013 | 34/34 | 63.4±8.7 | 62.2±7.9 | 16/18 | 19/15 | Early | BLC + BT | BT (Benazepril Hydrochloride) | 12 w | BLC 2.0g tid | Benazepril Hydrochloride (NR) | (2)(3) |
| Yang CH 2013 | 20/20 | 50.8±7.3 | 49.1±7.9 | 10/10 | 11/9 | Ⅲ | JSBC + BT | BT (Losartan) | 12 w | JSBC 3 capsules tid  (0.33g/capsule) | Losartan 50mg qd | (1)(2)(4)(7)(8) |
| Yu HT 2013 | 40/40 | Total 47.6±3.2 | | Total 52/28 | | Ⅲ | JSBC + BT | BT (Olmesartan Medoxomil) | 12 w | JSBC 3 capsules tid  (0.33g/capsule) | Olmesartan Medoxomil 20mg qd | (3)(5) |
| Zhou X 2013 | 72/65 | Total 46.2±7.3 | | Total 74/63 | | NR | JSBC + BT | BT (Benazepril) | 12 w | JSBC 6 capsules tid | Benazepril (NR) | (3)(7) |
| Ding T 2014 | 50/50 | Total 51.2±9.5 | | Total 52/48 | | NR | JSBC + BT | BT (Irbesartan) | 4 m | JSBC 3 capsules tid  (0.33g/capsule) | Irbesartan 75mg qd | (2)(3)(4) |
| Long YT 2014 | 60/60 | 48.3±6.6 | 47.3±6.8 | 35/25 | 38/22 | Ⅱ | BLC + BT | BT (Enalapril Maleate) | NR | BLC 1.0g tid | Enalapril Maleate 5mg (NR) | (2)(7)(8) |
| Lv XQ 2014 | 42/20 | 53.8±9.3 | 50.5±8.7 | 23/19 | 11/9 | NR | JSBC + BT | BT (Enalapril) | 6 m | JSBC 3 capsules tid | Enalapril 10mg bid | (6) |
| Wu L 2014 | 30/30 | 57.2±10.1 | 57.4±9.8 | 17/13 | 16/14 | Ⅲ | BLC + BT | BT (Valsartan) | 12 w | BLC 2.0g tid  (0.5g/capsule) | Valsartan 160mg qd | (1)(2)(8) |
| Zeng JF 2014 | 30/30 | 55.9±8.64 | 54.6±9.18 | 14/16 | 13/17 | Ⅲ-Ⅳ | BLC + BT | BT (Irbesartan) | 12 w | BLC 1.0g tid | Irbesartan 150mg qd | (2)(5)(6)(7)(8)(9)(10) |
| Zhang C 2014 | 40/39 | Total 50.4±10.2 | | Total 69/53 | | NR | JSBC + BT | BT (Losartan Potassium) | 8 w | JSBC 3 capsules tid | Losartan Potassium  50mg qd | (1)(2) |
| Zhang ZY 2014 | 30/30 | Total 64±6.3 | | Total 64/56 | | Ⅲ | JSBC + BT | BT (Candesartan Cilexetil) | 8 w | JSBC 3 capsules tid | Candesartan Cilexetil  (4mg/capsule) 4mg qd | (2)(4) |
| Zong L 2014 | 50/50 | 62.48±10.91 | 61.22±10.64 | 27/23 | 27/23 | NR | BLC + BT | BT (Losartan Potassium) | 180 d | BLC 1.0g tid  (0.2g/capsule) | Losartan Potassium 50mg qd | (2)(4)(6) |
| Cao XC 2015 | 45/45 | 57.8±8.0 | 57.4±6.1 | 31/14 | 30/15 | Ⅲ | BLC + BT | BT (Valsartan) | 16 w | BLC 1.0g tid | Valsartan 80mg qd | (1)(2)(4)(6)(7) |
| Diao L 2015 | 45/45 | 57.8±6.0 | 57.4±6.1 | 31/14 | 30/15 | Ⅲ | BLC + BT | BT (Valsartan) | 16 w | BLC 1.0g tid | Valsartan 80mg qd | (1)(2)(4)(6)(7) |
| Wang XH 2015 | 50/50 | 53.1±2.3 | 52.3±2.5 | 26/24 | 27/23 | NR | JSBC + BT | BT (Benazepril Hydrochloride) | 4 w | JSBC 3 capsules tid  (0.33g/capsule) | Benazepril Hydrochloride  (5mg/capsule) 5mg qd | (1)(2)(3)(4)(6) |
| Xu L 2015 | 50/50 | 50.9±5.1 | 52.1±4.9 | 30/20 | 29/21 | Early | JSBC + BT | BT (Irbesartan) | 20 w | JSBC 3 capsules qid | Irbesartan 150mg qd | (1) |
| Zen XM 2015 | 92/92 | 38.1±11.5 | 39.6±10.7 | 49/43 | 47/45 | NR | JSBC + BT | BT (Valsartan) | 6 m | JSBC 3 capsules tid | Valsartan 80mg qd | (2)(3)(6)(7)(8)(10) |
| Zhou XY 2015 | 20/20 | 63.1±2.8 | 62.6±2.5 | 12/8 | 11/9 | Early | ZLC + BT | BT (Valsartan) | 12 w | ZLC 2 capsules tid | Valsartan 80mg qd | (1)(2)(4) |
| Zhu J 2015 | 30/30 | 40.9±7.6 | 40.6±7.5 | 15/15 | 16/14 | Early | JSBC + BT | BT (Valsartan) | NR | JSBC 3 capsules tid  (0.33g/capsule) | Valsartan 80mg qd | (3)(6) |
| Chen QS 2016 | 35/35 | 62.3±4.7 | 63.4±5.0 | 17/18 | 19/16 | Early | BLC + BT | BT (Benazepril) | 12 w | BLC tid  (0.5g/capsule) | Benazepril 10mg qd | (1)(2)(4)(6) |
| Dai HH 2016 | 45/45 | 52±7.5 | 51±7.5 | 30/15 | 34/11 | Early | JSBC + BT | BT (Valsartan) | 7 m | JSBC 3 capsules tid | Valsartan 80~160mg qd | (1)(2)(3)(8) |
| Huang JY 2016 | 58/60 | 58.21±5.18 | 58.87±6.02 | 32/26 | 31/29 | Early | BLC + BT | BT (Losartan) | 3 m | BLC 2.0g bid  (0.5g/capsule) | Losartan 50mg qd | (1)(7)(8) |
| Hu Y 2016 | 80/80 | Total 39~73 | | Total 87/73 | | NR | BLC + BT | BT (Irbesartan) | 3 m | BLC 0.4g tid | Irbesartan 150mg qd | (2)(3) |
| Jin XB 2016 | 50/50 | 55.3±9.8 | 54.1±10.1 | 26/24 | 28/22 | Ⅲ | BLC + BT | BT (Valsartan) | 12 w | BLC 8 capsules tid | Valsartan 80mg qd | (2)(3)(4)(7) |
| Lin ZN 2016 | 40/40 | Total 50~70 | | 20/20 | 20/20 | Early | BLC + BT | BT (Benazepril) | 12 w | BLC 1.0g tid | Benazepril 10mg qd | (2)(4)(6) |
| Liu CY 2016(1) | 32/28 | 50.67±7.41 | 51.24±7.32 | 20/12 | 17/11 | Ⅲ-Ⅳ | BLC + BT | BT (Irbesartan) | 12 w | BLC 3.0g tid | Irbesartan 150mg qd | (1)(2)(4) |
| Liu CY 2016(2) | 32/28 | 51±7 | 51±7 | 20/12 | 17/11 | Ⅲ-Ⅳ | BLC + BT | BT (Irbesartan) | 12 w | BLC 3.0g tid | Irbesartan 150mg qd | (7)(8) |
| Pan J 2016 | 40/40 | 64.5±4.7 | 65.7±5.2 | 28/12 | 24/16 | Ⅲ | JSBC + BT | BT (Telmisartan) | 3 m | JSBC 3 capsules tid | Telmisartan 80mg qd | (3)(4)(5) |
| Tang XD 2016 | 40/40 | Total 56.3±0.2 | | Total 45/35 | | Early | BLC + BT | BT (Irbesartan) | 16 w | BLC 2.0g tid | Irbesartan 150mg qd | (3)(5)(7)(8) |
| Wu QF 2016 | 34/34 | 52.12±3.23 | 50.56±4.12 | 18/16 | 22/12 | Ⅲ | JSBC + BT | BT (Candesartan Cilexetil) | 8 w | JSBC 3 capsules tid | Candesartan Cilexetil 4mg qd | (1)(2)(4) |
| Xiu CT 2016 | 55/55 | 53.2±6.8 | 52.8±5.7 | 28/27 | 26/29 | Early | JSBC + BT | BT (Candesartan) | 3 m | JSBC 3 capsules tid | Candesartan 4mg qd | (6)(8) |
| Yang G 2016 | 50/50 | 54.2±9.5 | 53.0±9.2 | 21/29 | 23/27 | NR | BLC + BT | BT (Irbesartan) | 3 m | BLC 5 capsules tid | Irbesartan  (0.15 g/capsule)  1~2capsules qd | (1)(2)(3)(4)(7)(8) |
| Zhang LH 2016 | 33/34 | 56.4±3.3 | 55.6±4.1 | 15/18 | 18/16 | Ⅲ | JSBC + BT | BT (Irbesartan) | 3 m | JSBC 4 capsules qid | Irbesartan 150mg qd | (1)(2)(8) |
| Zhan J 2016 | 62/48 | 56.76±6.51 | 55.76±5.28 | 35/27 | 27/21 | Early | BLC + BT | BT (Enalapril) | 90 d | BLC 1.0g tid  (0.5g/capsule) | Enalapril 5mg qd | (1)(2)(8)(10) |
| Chen QJ 2017 | 60/60 | Total 66.5±2.7 | | Total 62/58 | | Ⅰ-Ⅲ | JSBC + BT | BT (Losartan Potassium) | NR | JSBC 3 capsules tid | Losartan Potassium 50mg qd | (2)(4) |
| Feng ZL 2017 | 43/43 | 47.02±2.06 | 46.92±2.11 | 23/20 | 24/19 | Early | JSBC + BT | BT (Valsartan) | 4 w | JSBC 3 capsules tid  (0.33g/capsule) | Valsartan 80~160mg qd | (1)(3)(8) |
| He XJ 2017 | 67/67 | 55.79±6.58 | 56.13±6.44 | 37/30 | 36/31 | Early | BLC + BT | BT (Valsartan) | NR | BLC 4 capsules tid | Valsartan 80mg qd | (2)(3)(4)(7) |
| Hou K 2017 | 30/30 | 57.2±3.0 | 56.7±2.8 | 10/20 | 15/15 | Early | BLC + BT | BT (Valsartan) | 16 w | BLC 1.0g tid | Valsartan 80mg qd | (2)(4)(6)(7) |
| Lei GY 2017 | 38/38 | Total 60.8±5.4 | | Total 42/34 | | NR | BLC + BT | BT (Telmisartan) | 3 m | BLC 2.0g tid | Telmisartan 80mg qd | (1) |
| Liu WY 2017 | 41/41 | Total 63.3±5.7 | | Total 43/39 | | Early | JSBC + BT | BT (Valsartan) | 8 w | JSBC 3 capsules tid  (0.33g/capsule) | Valsartan 80mg qd | (2)(4)(7)(8) |
| Liu YX 2017 | 61/61 | 61.45±9.36 | 61.51±9.55 | 42/19 | 36/25 | Early | ZLC + BT | BT (Valsartan) | 3 m | ZLC 2 capsules tid | Valsartan 80mg qd | (2)(9) |
| Li W 2017 | 46/45 | 60.2±5.1 | 62.3±6.5 | 30/16 | 32/13 | Early | JSBC + BT | BT (Benazepril Hydrochloride) | 8 w | JSBC 3 capsules tid | Benazepril Hydrochloride 10mg qd | (1)(2)(3)(4)(6) |
| Ma J 2017 | 30/30 | 69.13±8.72 | 69.25±8.26 | 17/13 | 18/12 | NR | JSBC + BT | BT (Irbesartan) | 2 m | JSBC 6 capsules tid  (0.33g/capsule) | Irbesartan 150mg qd | (2)(3)(7) |
| Song XP 2017 | 20/20 | Total 40~68 | | Total 32/28 | | Ⅲ-Ⅳ | JSBC + BT | BT (Benazepril) | 6 m | JSBC 3capsules tid | Benazepril 10mg qd | (1)(2)(4)(5)(6) |
| Yang T 2017 | 49/49 | 52.61±2.13 | 54.38±2.67 | 21/28 | 26/23 | NR | BLT + BT | BT (Candesartan) | 1 m | BLT 1 capsule bid  (0.45g/capsule) | Candesartan 8mg qd | (2)(4)(6)(7)(8) |
| Yuan YH 2017 | 51/51 | 53.3±2.6 | 53.9±2.4 | 28/23 | 25/26 | Early | BLC + BT | BT (Telmisartan) | 3 m | BLC 9.0g qd | Telmisartan 80mg qd | (2)(3)(4) |
| Zhang W 2017 | 62/62 | 48~77 | 49~77 | 33/29 | 35/27 | NR | JSBC + BT | BT (Valsartan) | 8 w | JSBC 6 capsules tid  (0.33g/capsule) | Valsartan 80mg qd | (2)(4) |
| Gao X 2018 | 76/76 | 67.19±5.21 | 67.20±5.34 | 49/27 | 50/26 | NR | JSBC + BT | BT (Losartan Potassium) | 45 d | JSBC 3 capsules tid | Losartan Potassium  50mg qd | (2)(7)(8) |
| Hu Y 2018 | 60/60 | 49.2±4.8 | 47.6±5.3 | 32/28 | 29/31 | Ⅲ | JSBC + BT | BT (Valsartan) | 24 w | JSBC 3 capsules tid  (0.33g/capsule) | Valsartan 80mg qd | (1)(2)(3)(4)(6)(7)(8) |
| Luo JG 2018 | 31/31 | 53.61±12.86 | 54.75±10.69 | 14/17 | 13/18 | Ⅲ | BLC + BT | BT (Irbesartan) | 3 m | BLC 1.0g tid | Irbesartan 150mg qd | (1)(2)(3)(4)(7) |
| Ma JL 2018 | 50/50 | 64.57±12.24 | 64.83±12.31 | 28/22 | 29/21 | NR | JSBC + BT | BT (Benazepril Hydrochloride) | NR | JSBC 3 capsules tid  (0.33g/capsule) | Benazepril Hydrochloride 5mg qd | (1)(2)(3)(4)(6) |
| Shen XY 2018 | 30/30 | 58.2±4.5 | 59.1±4.3 | 12/18 | 17/13 | Ⅲ | JSBC + BT | BT (Irbesartan) | 3 m | JSBC 3 capsules tid | Irbesartan 150mg qd | (1)(2)(4)(7)(8) |
| Wang W 2018 | 48/50 | 54.7±5.6 | 52.9±4.7 | 23/25 | 29/21 | Ⅱ-Ⅳ | ZLC + BT | BT (Telmisartan) | 12 w | ZLC 2 capsules tid | Telmisartan  1 capsule qd | (2)(3)(6)(8) |
| Xu ZM 2018 | 30/30 | 56.1±7.5 | 54.6±6.8 | 8/22 | 10/20 | Early | JSBC + BT | BT (Benazepril Hydrochloride) | 3 m | JSBC 3 capsules tid | Benazepril Hydrochloride 5mg qd | (1)(2)(3)(4) |
| Dai GJ 2019 | 51/51 | 60.77±8.48 | 60.05±9.13 | 27/24 | 29/22 | NR | JSBC + BT | BT (Irbesartan) | 3 m | JSBC 3 capsules tid  (0.33g/capsule) | Irbesartan 150mg qd | (1)(2)(3)(4)(6) |
| Hu XJ 2019 | 50/50 | 55.64±5.42 | 55.36±5.28 | 32/18 | 29/21 | Early | JSBC + BT | BT (Benazepril) | 3 m | JSBC 3 capsules tid | Benazepril 5mg qd | (1)(2)(3)(4) |
| Li N 2019 | 20/20 | 64.28±3.02 | 63.86±2.94 | Total 21/19 | | Early | BLC + BT | BT (Irbesartan) | 8 w | BLC 4 capsules tid | Irbesartan 150mg qd | (3)(7) |
| Liu HY 2019 | 56/56 | 46.2±5.2 | 45.2±4.7 | 28/28 | 26/30 | NR | JSBC + BT | BT (Benazepril Hydrochloride) | 24 w | JSBC 3 capsules tid  (0.33g/capsule) | Benazepril Hydrochloride 5mg qd | (1)(2)(3)(4)(6) |
| Liu MJ 2019 | 49/49 | 54.15±4.92 | 55.32±5.17 | 21/28 | 25/24 | Early | BLC + BT | BT (Ramipril) | 2 m | BLC 1.0g tid  (0.5g/capsule) | Ramipril 5mg tid | (1)(3)(7)(8) |
| Li Z 2019 | 71/71 | 53~71 | 51~72 | 35/36 | 37/34 | Ⅲ | BLC + BT | BT (Irbesartan) | 8 w | BLC 5 capsules tid | Irbesartan 150mg qd | (1)(2)(4) |
| Qiu LF 2019 | 48/48 | 44.82±2.06 | 50.24±2.84 | 26/22 | 23/25 | Early | BLC + BT | BT (Candesartan Cilexetil) | 3 m | BLC 9.0g tid | Candesartan Cilexetil 4mg qd | (1)(2) |
| Tian XY 2019 | 41/41 | 67.12±6.47 | 66.48±6.52 | Total 58/24 | | Early | BLC + BT | BT (Valsartan) | 3 m | BLC 8 capsules tid | Valsartan 80mg qd | (2)(3)(4)(6) |
| Wu HJ 2019 | 41/41 | 58.93±6.42 | 59.18±6.17 | 26/15 | 27/14 | Ⅲ-Ⅳ | JSBC + BT | BT (Irbesartan) | 8 w | JSBC 6 capsules tid | Irbesartan 150mg qd | (7)(10) |
| Yang R 2019 | 32/32 | 52.6±8.2 | 53.2±8.8 | 22/10 | 18/14 | NR | BLC + BT | BT (Valsartan) | 12 w | BLC 1.0g tid | Valsartan 80mg qd | (1)(2)(4)(7) |
| Zhang YY 2019 | 35/35 | 51.17±6.96 | 50.34±6.57 | 16/19 | 18/17 | NR | JSBC + BT | BT (Irbesartan) | 8 w | JSBC 1.98g tid | Irbesartan 150mg qd | (1)(2)(4)(6) |
| Liu J 2020 | 30/30 | 57.89±5.12 | 58.69±4.53 | 16/14 | 18/12 | Ⅲ | JSBC + BT | BT (Losartan Potassium) | 2 m | JSBC 3 capsules tid  (0.33g/capsule) | Losartan Potassium  50mg qd | (2)(8) |
| Lu JY 2020 | 71/71 | 61.1±5.2 | 60.3±5.0 | Total 79/63 | | NR | BLC + BT | BT (Perindopril) | 3 m | BLC 1.0g tid | Perindopril 8mg qd | (2)(4)(7)(8) |
| Ren X 2020 | 126/126 | 62.8±5.4 | 62.6±5.5 | 65/61 | 66/60 | Early | BLC + BT | BT (Irbesartan) | 3 m | BLC 1.0g tid | Irbesartan 150mg qd | (6) |
| Ren YF 2020 | 60/60 | 49.8±5.2 | 50.3±4.9 | Total 68/52 | | Ⅱ-Ⅲ | BLC + BT | BT (Valsartan + Simvastatin) | 3 m | BLC 1.0g tid | Valsartan (NR) + Simvastatin 40mg qd | (1)(2)(4)(6)(7)(8) |
| Wang AY 2020 | 39/39 | 64.89±5.82 | 64.51±5.69 | 20/19 | 21/18 | Early | JSBC + BT | BT (Candesartan Cilexetil) | 8 w | JSBC 3 capsules tid  (0.33g/capsule) | Candesartan Cilexetil 4mg qd | (2)(4)(8) |
| Wang KY 2020 | 30/30 | 49.5±5.8 | 49.6±5.9 | Total 35/25 | | NR | BLC + BT | BT (Valsartan) | 60 d | BLC 3 capsules tid | Valsartan 80mg qd | (1)(6)(7)(8) |
| Wang YD 2020 | 34/34 | 62.21±2.65 | 56.11±4.13 | 19/15 | 18/14 | Early | BLC + BT | BT (Benazepril) | 12 w | BLC 1.0g tid  (0.5g/capsule) | Benazepril 10mg qd | (1)(2) |
| Cheng J 2021 | 26/26 | 55.46±8.53 | 55.66±8.75 | 16/10 | 14/12 | NR | BLC + BT | BT (Irbesartan) | 3 m | BLC 1.0g tid | Irbesartan 150mg qd | (2)(4)(6)(7) |
| Du N 2021 | 40/40 | 71.08±2.55 | 72.16±2.79 | 24/16 | 23/17 | Ⅲ-Ⅳ | BLC + BT | BT (Irbesartan) | 3 m | BLC 1.0g tid | Irbesartan 75~300mg qd | (1)(2)(7)(8) |
| Guan CA 2021 | 42/42 | 51.91±7.72 | 52.29±9.06 | 28/14 | 26/16 | Ⅱ-Ⅲ | BLC + BT | BT (Candesartan Cilexetil) | 2 w | BLC 4 capsules tid  (0.5g/capsule) | Candesartan Cilexetil 4~12mg | (1)(2)(3)(4)(6) |
| Han PP 2021 | 43/43 | 57.11±4.56 | 56.82±4.73 | 21/22 | 22/21 | NR | JSBC + BT | BT (Irbesartan) | 8 w | JSBC 6 capsules tid | Irbesartan 150mg qd | (7) |
| Jiang T 2021 | 69/69 | 57.74±5.32 | 56.87±5.81 | 41/28 | 38/31 | Ⅲ | JSBC + BT | BT (Ramipril) | 2 m | JSBC 6 capsules tid | Ramipril 2.5mg qd | (1)(2)(3)(4)(6) |
| Pan X 2021 | 39/39 | 69.89±5.17 | 69.64±5.23 | 24/15 | 26/13 | NR | JSBT + BT | BT (Irbesartan) | 3 m | JSBT 3 capsules tid | Irbesartan 150mg qd | (2)(4)(6)(7)(8) |
| Shen LP 2021 | 40/40 | 67.16±4.46 | 67.08±5.18 | 25/15 | 23/17 | Ⅲ | BLT + BT | BT (Benazepril) | 12 w | BLT 0.9g tid | Benazepril 5~20mg qd | (2)(4)(6) |
| Xiao Y 2021 | 67/67 | 63.87±4.98 | 64.31±4.92 | 37/30 | 35/32 | Early | BLC + BT | BT (Irbesartan) | NR | BLC 1.0g tid | Irbesartan 150mg qd | (1)(2)(4)(6) |
| Xie M 2021 | 44/44 | 59.55±4.33 | 59.3±4.77 | 25/19 | 26/18 | NR | JSBC + BT | BT (Irbesartan) | 3 m | JSBC 3 capsules tid | Irbesartan 150mg qd | (3) |
| Xu YY 2021 | 43/43 | 57.12±6.31 | 56.23±5.15 | 28/15 | 26/17 | Early | JSBC + BT | BT (Losartan Potassium) | 12 w | JSBC 3 capsules tid  (0.33g/capsule) | Losartan Potassium  50mg qd | (2)(4)(7)(8) |
| Yu WH 2021 | 80/80 | 49.97±10.65 | 50.13±11.26 | 39/41 | 37/43 | NR | BLC + BT | BT (Losartan) | 1 m | BLC 5 capsules tid | Losartan 50mg qd | (2)(3)(4)(6)(9) |
| Zhang Y 2021 | 84/84 | 50.90±4.71 | 50.85±4.65 | 50/34 | 46/38 | Ⅲ | JSBC + BT | BT (Irbesartan) | 3 m | JSBC 3 capsules tid  (0.33g/capsule) | Irbesartan 150mg qd | (2)(4) |
| Ding L 2022 | 50/48 | 69.14±6.82 | 68.81±5.96 | 26/24 | 25/23 | Ⅲ | BLC + BT | BT (ACEI/ARB) | 8 w | BLC 5capsules tid  (0.5g/capsule) | ACEI/ARB (NR) | (1)(2)(4)(10) |
| Dong YQ 2022 | 30/30 | 55.3±8.5 | 56.7±7.2 | 12/18 | 16/14 | Ⅲ-Ⅳ | BLC + BT | BT (Irbesartan) | 8 w | BLC 0.4~1.2g tid  (0.2g/capsule) | Irbesartan 150mg qd | (2)(4) |
| Huang D 2022 | 58/58 | 68.12±4.04 | 68.76±4.27 | 32/26 | 30/28 | Ⅲ | BLC + BT | BT (Candesartan Cilexetil) | 8 w | BLC 5capsules tid  (0.2g/capsule) | Candesartan Cilexetil 8mg qd | (1)(2)(3)(4)(7)(8) |
| Lv WQ 2022 | 40/40 | 62.15±2.16 | 61.96±2.14 | 25/15 | 23/17 | NR | JSBT + BT | BT (Irbesartan) | 3 m | JSBT 5 capsules tid | Irbesartan 150mg qd | (1)(2)(4)(7)(8) |
| Yu XH 2022 | 46/46 | 65.11±6.12 | 64.72±6.02 | Total 51/41 | | Early | BLC + BT | BT (Benazepril Hydrochloride) | 12 w | BLC 1.5g tid | Benazepril Hydrochloride 10mg qd | (1)(2)(3)(6) |
| Li HS 2023 | 47/47 | 60.24±8.73 | 59.62±8.45 | 28/19 | 25/22 | Ⅲ | JSBT + BT | BT (Irbesartan) | 12 w | JSBT 3 tablets tid | Irbesartan 150mg qd | (1)(2)(4)(6) |

**Note：**(1) Urinary albumin excretion rate; (2) Serum creatinine; (3)24 hours urinary total protein; (4) Serum urea nitrogen; (5) Urinary albumin/creatinine ratio; (6) Adverse events; (7) Fasting plasma glucose; (8) Glycated hemoglobin A1c; (9) Estimated glomerular filtration rate.

**Abbreviations:** E, experimental group; C, control group; DKD, diabetic kidney disease; BT, basic treatment; NR, not reported; BLC, Bailing capsule; BLT, Bailing tablet; JSBC, Jinshuibao capsule; JSBT, Jinshuibao tablet; ZLC, Zhiling capsule; m, month(s); w, week(s); d, days; qd, every day; tid, three times a day.
