# Supplementary material for: Ophiocordyceps sinensis preparations combined with the renin–angiotensin system inhibitor for diabetic kidney disease treatment: an umbrella review of systematic reviews and network meta-analysis
Source: Front Pharmacol. 2024 Apr 22;15:1360633. doi: 10.3389/fphar.2024.1360633 (PMC11075507; doi:10.3389/fphar.2024.1360633)
Supplement: Supplementary file 9 [file Table3.DOCX]

Supplementary Material

*Ophiocordyceps sinensis* preparations combined with renin-angiotensin system inhibitor for diabetic kidney disease: an umbrella review of systematic reviews and network meta-analysis

**Xue Xue^1^****^†^, Xin-yan Jin^2†^, Xing-lan Ye^3^, Ke-ying Li^3^, Jia-xuan Li^3^, Xue-han Liu^2^, Juan Bai^3^, Qiang Liu^4^, Bing-rui Zhang^5^, Xin-rong Zou^4^, Jun Yuan^6^, Chun-li Lu^7^, Fang-fang Zhao^8^, Jian-ping Liu^2^* and Xiao-qin Wang^4^***

*** Correspondence:**Jian-ping Liu: Liujp@bucm.edu.cn

Xiao-qin Wang: wangxiaoqin773@hotmail.com

# Supplementary Table 3 Methodological quality evaluation of the included systematic reviews (AMSTAR 2)

| **Study ID** | **Item1** | **Item2*** | **Item3** | **Item4*** | **Item5** | **Item6** | **Item7*** | **Item8** | **Item9*** | **Item10** | **Item**  **11*** | **Item**  **12** | **Item13*** | **Item14** | **Item15*** | **Item**  **16** | **Overall quality** |
| --- | --- | --- | --- | --- | --- | --- | --- | --- | --- | --- | --- | --- | --- | --- | --- | --- | --- |
| Tang L 2013 | Y | N | Y | PY | Y | Y | N | PY | Y | N | Y | Y | Y | N | Y | N | Critically low |
| Duan R 2015 | Y | N | Y | PY | Y | Y | N | Y | Y | N | Y | Y | Y | N | Y | N | Critically low |
| Luo Y 2015 | Y | N | Y | PY | Y | Y | N | Y | Y | N | Y | N | N | Y | Y | Y | Critically low |
| Cai T 2016 | Y | N | Y | PY | Y | Y | N | Y | Y | N | Y | Y | Y | Y | Y | N | Critically low |
| Li GD 2016 | Y | N | Y | PY | N | N | N | Y | N | N | Y | N | N | N | N | N | Critically low |
| Zhang Y 2018 | Y | N | Y | Y | Y | Y | N | Y | Y | N | Y | Y | Y | Y | Y | N | Critically low |
| Lu Q 2018 | Y | N | Y | PY | Y | Y | N | PY | Y | N | Y | Y | Y | Y | Y | Y | Critically low |
| Huang YL 2019 | Y | N | Y | PY | Y | Y | N | Y | Y | N | Y | Y | Y | N | Y | N | Critically low |
| Lian XY 2020 | Y | N | Y | PY | Y | Y | N | Y | Y | N | Y | Y | Y | N | Y | N | Critically low |
| Li YB 2019 | Y | N | Y | PY | Y | Y | N | Y | Y | N | Y | Y | Y | Y | Y | Y | Critically low |
| Gong L 2021 | Y | N | Y | PY | Y | Y | N | PY | Y | N | Y | Y | Y | Y | Y | N | Critically low |
| Zhou XM 2021 | Y | N | Y | PY | Y | Y | N | N | Y | N | Y | Y | Y | Y | Y | Y | Critically low |
| Li XJ 2022 | Y | N | Y | Y | Y | Y | N | Y | Y | N | Y | Y | Y | N | Y | N | Critically low |
| Yan GC 2023 | Y | Y | Y | Y | Y | Y | Y | Y | Y | N | Y | Y | Y | Y | Y | Y | High |
|  |  |  |  |  |  |  |  |  |  |  |  |  |  |  |  |  |  |
| **Item** | **1** | **2*** | **3** | **4*** | **5** | **6** | **7*** | **8** | **9*** | **10** | **11*** | **12** | **13*** | **14** | **15*** | **16** |  |
| **Y** | 14 | 1 | 14 | 3 | 13 | 13 | 1 | 10 | 13 | 0 | 14 | 12 | 12 | 8 | 13 | 5 |  |
| **PY** | 0 | 0 | 0 | 11 | 0 | 0 | 0 | 3 | 0 | 0 | 0 | 0 | 0 | 0 | 0 | 0 |  |
| **N** | 0 | 13 | 0 | 0 | 1 | 1 | 13 | 1 | 1 | 14 | 0 | 2 | 2 | 6 | 1 | 9 |  |

**Note:** *****The critical items of AMSTAR-2; Y, yes; PY, partial yes.

Item1: Did the research questions and inclusion criteria for the review include the components of PICO?

Item2: Did the report of the review contain an explicit statement that the review methods were established prior to the conduct of the review and did the report justify any significant deviations from the protocol?

Item3: Did the review authors explain their selection of the study designs for inclusion in the review?

Item4: Did the review authors use a comprehensive literature search strategy?

Item5: Did the review authors perform study selection in duplicate?

Item6: Did the review authors perform data extraction in duplicate?

Item7: Did the review authors provide a list of excluded studies and justify the exclusions?

Item8: Did the review authors describe the included studies in adequate detail?

Item9: Did the review authors use a satisfactory technique for assessing the risk of bias (RoB) in individual studies that were included in the review?

Item10: Did the review authors report on the sources of funding for the studies included in the review?

Item11: If meta-analysis was performed did the review authors use appropriate methods for statistical combination of results?

Item12: If meta-analysis was performed, did the review authors assess the potential impact of RoB in individual studies on the results of the meta-analysis or other

evidence synthesis?

Item13: Did the review authors account for RoB in individual studies when interpreting/discussing the results of the review?

Item14: Did the review authors provide a satisfactory explanation for, and discussion of, any heterogeneity observed in the results of the review?

Item15: If they performed quantitative synthesis did the review authors carry out an adequate investigation of publication bias (small study bias) and discuss its likely

impact on the results of the review?

Item16: Did the review authors report any potential sources of conflict of interest, including any funding they received for conducting the review?
