# Supplementary material for: Ophiocordyceps sinensis preparations combined with the renin–angiotensin system inhibitor for diabetic kidney disease treatment: an umbrella review of systematic reviews and network meta-analysis
Source: Front Pharmacol. 2024 Apr 22;15:1360633. doi: 10.3389/fphar.2024.1360633 (PMC11075507; doi:10.3389/fphar.2024.1360633)
Supplement: Supplementary file 10 [file Table4.DOCX]

Supplementary Material

*Ophiocordyceps sinensis* preparations combined with renin-angiotensin system inhibitor for diabetic kidney disease: an umbrella review of systematic reviews and network meta-analysis

**Xue Xue^1^****^†^, Xin-yan Jin^2†^, Xing-lan Ye^3^, Ke-ying Li^3^, Jia-xuan Li^3^, Xue-han Liu^2^, Juan Bai^3^, Qiang Liu^4^, Bing-rui Zhang^5^, Xin-rong Zou^4^, Jun Yuan^6^, Chun-li Lu^7^, Fang-fang Zhao^8^, Jian-ping Liu^2^* and Xiao-qin Wang^4^***

*** Correspondence:**Jian-ping Liu: Liujp@bucm.edu.cn

Xiao-qin Wang: wangxiaoqin773@hotmail.com

# Supplementary Table 4 Summary of replies by authors

| **Study ID** | **Questionable information in the original study** | **Respond** |
| --- | --- | --- |
| Qiu LF 2019 | 1. Nonexistent name of intervention patent.   2.Incorrect unit of Scr. | The author confirmed that the name of patent and the unit were written errors. (By phone) |
| Liu MW 2011 | 1.Nonexistent name of intervention patent. | The author confirmed that the name of patent was written error. (By phone) |
| Li QY 2012 | 1.Incorrect unit of Scr. | The author confirmed that the unit was written error. (By phone) |

**Abbreviations:** Scr, serum creatinine.
