# Supplementary material for: Ophiocordyceps sinensis preparations combined with the renin–angiotensin system inhibitor for diabetic kidney disease treatment: an umbrella review of systematic reviews and network meta-analysis
Source: Front Pharmacol. 2024 Apr 22;15:1360633. doi: 10.3389/fphar.2024.1360633 (PMC11075507; doi:10.3389/fphar.2024.1360633)
Supplement: Supplementary file 11 [file Table6.DOCX]

Supplementary Material

*Ophiocordyceps sinensis* preparations combined with renin-angiotensin system inhibitor for diabetic kidney disease: an umbrella review of systematic reviews and network meta-analysis

**Xue Xue^1^****^†^, Xin-yan Jin^2†^, Xing-lan Ye^3^, Ke-ying Li^3^, Jia-xuan Li^3^, Xue-han Liu^2^, Juan Bai^3^, Qiang Liu^4^, Bing-rui Zhang^5^, Xin-rong Zou^4^, Jun Yuan^6^, Chun-li Lu^7^, Fang-fang Zhao^8^, Jian-ping Liu^2^* and Xiao-qin Wang^4^***

*** Correspondence:**Jian-ping Liu: Liujp@bucm.edu.cn

Xiao-qin Wang: wangxiaoqin773@hotmail.com

# Supplementary Table 6 Adverse events of experimental group and control group

| ***OS* preparations** | **Adverse events** | **Experimental group:**  ***OS* with ACEIs/ARBs**  **(967 patients)** | **Control group:**  **ACEIs/ARBs  (960 patients)** |
| --- | --- | --- | --- |
| BLC | Gastrointestinal discomfort | 15（1.55%） | 12（1.25%） |
|  | Liver function injury | 1（0.10%） | 0（0.00%） |
|  | Renal function injury | 1（0.10%） | 0（0.00%） |
|  | Mild edema of ankle and wrist | few | few |
|  | Rash | 2（0.21%） | 1（0.10%） |
|  | Headache and dizziness | 4（0.41%） | 13（1.35%） |
|  | Pharyngeal discomfort | 2（0.21%） | 5（0.52%） |
|  | Decreased hemoglobin | 2（0.21%） | 1（0.10%） |
| JSBC | Gastrointestinal discomfort | 7（0.72%） | 8（0.83%） |
|  | Liver function injury | 2（0.21%） | 4（0.42%） |
|  | Renal function injury | 3（0.31%） | 3（0.31%） |
|  | Rash | 0（0.00%） | 1（0.10%） |
|  | Headache and dizziness | 9（0.93%） | 8（0.83%） |
|  | Dry cough | 4（0.41%） | 5（0.52%） |
|  | Hypoglycemia | 4（0.41%） | 3（0.31%） |
|  | Drowsiness | 2（0.21%） | 0（0.00%） |
|  | Palpitations | 4（0.41%） | 3（0.31%） |
|  | Vasculitis | 0（0.00%） | 5（0.52%） |
| BLT | Gastrointestinal discomfort | 6（0.62%） | 7（0.73%） |
|  | Hypoglycemia | 1（0.10%） | 2（0.21%） |
|  | Rash | 1（0.10%） | 1（0.10%） |

**Abbreviation:** *OS*, *Ophiocordyceps sinensis*; BLC, Bailing capsule; JSBC, Jinshuibao capsule; BLT, Bailing tablet; ACEIs/ARBs, angiotensin converting enzyme inhibitors/angiotensin receptor blockers.
